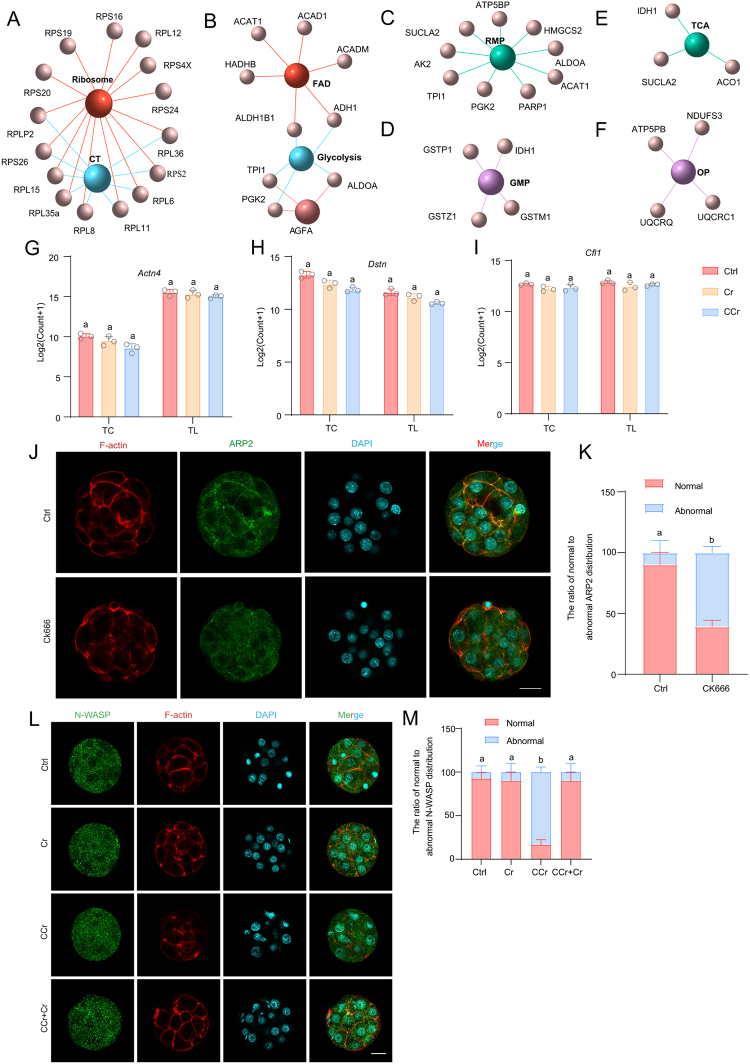# Creatine metabolism regulates trophectoderm formation in early mouse embryos *via* an energy-cytoskeleton-YAP axis

**DOI:** 10.1016/j.jbc.2026.113147

**Published:** 2026-05-18

**Authors:** Xiangyi Chen, Bo Pan, Jianpeng Qin, Yaozong Wei, Kunlin Du, Ao Ning, Tao Sun, Can Wang, Shuqi Zou, Xiaohui Su, Mengying Wang, Yinan Guo, Xiaoyue Xiong, Shiyuan Pei, Shengqin Zang, Chang Zhang, Jiangfeng Ye, Guozhi Yu, Qiuxia Liang, Guangbin Zhou

**Affiliations:** 1 State Key Laboratory of Swine and Poultry Breeding Industry, Key Laboratory of Livestock and Poultry Multiomics, Ministry of Agriculture and Rural Affairs, Farm Animal Genetic Resources Exploration and Innovation Key Laboratory of Sichuan Province, College of Animal Science and Technology, Sichuan Agricultural University, Chengdu, China; 2 College of Life Science, Sichuan Agricultural University, Ya'an, China

**Keywords:** creatine metabolism, trophectoderm formation, early embryonic development, YAP, cytoskeleton, energy metabolism

## Abstract

Creatine metabolism plays a well-established role in somatic cell energy homeostasis, but its function and mechanisms during early mammalian embryonic development remain unclear. Our findings reveal that creatine metabolism is critical for mouse embryo development from the 8-cell to the blastocyst stage. Inhibition of creatine metabolism causes stage-specific arrest at the morula-to-blastocyst transition, significantly impairing trophectoderm (TE) formation in blastocysts, and has no significant effect on the inner cell mass (ICM). Mechanistically, disruption of creatine metabolism leads to decreased ATP levels, which not only disturbs actin-related protein 2 (ARP2) distribution and inhibits ARP2-mediated assembly of F-actin but also activates AMPK. Together, these effects hinder the nuclear localization of the Hippo pathway effector Yes-associated protein (YAP). Impaired YAP nuclear translocation results in the downregulation of genes related to TE formation, actin cytoskeleton, and creatine metabolism. Exogenous supplementation with creatine, YAP agonists, or AMPK inhibitors can partially reverse the aforementioned abnormalities. This study uncovers that creatine metabolism regulates TE formation during early embryonic development through a central axis of “energy buffering–F-actin assembly–YAP activation,” providing new insights into the metabolic regulation of preimplantation development in mammals.

Mammalian early embryonic development initiates with the zygote and undergoes a series of critical biological events, including cleavage, compaction, polarization, and blastocyst formation. Among these, blastocyst formation marks the first lineage differentiation—the establishment of the ICM and TE (1, 2). This differentiation process is tightly regulated. Key aspects include metabolic reprogramming (3), establishment of cellular polarity (4), and integration of signaling pathways (5, 6, 7, 8, 9, 10, 11). The TE serves as the precursor of the placenta and is essential for embryo implantation and subsequent pregnancy maintenance (12). Although the core roles of the Hippo signaling pathway and its effector YAP in TE differentiation are established (13, 14, 15, 16), and the importance of a metabolic shift toward glycolysis for blastocyst development is well-recognized (17), how early embryos sense and integrate energy status to precisely regulate cell fate decisions, and what the upstream metabolic hubs and specific mechanisms remain poorly understood.

Metabolites are not only core substrates for energy production and biosynthesis but also key regulators of cell fate, capable of directly modulating gene expression (18, 19). Recent studies have demonstrated that metabolites play critical roles in embryonic development and cell fate determination. For instance, pyruvate and lactate support the zygotic genome activation (20), dynamic changes in L-2-hydroxyglutarate (L-2-HG) regulate epigenetic remodeling during the oocyte-to-embryo transition (21), lipid metabolism is involved in the establishment of apical polarity in outer blastomeres (22), and glucose metabolism-mediated regulation of YAP activity is essential for the differentiation of ICM and TE at the blastocyst stage (13). However, whether a central "metabolic master switch" exists that bridges energy metabolism, cytoskeletal dynamics, and transcriptional regulation remains to be explored.

As a key regulatory molecule in energy metabolism, creatine maintains the ATP/ADP ratio within cells through the creatine/phosphocreatine system, providing an efficient and immediate energy buffer for processes with high-energy demands (23, 24). The role of creatine in tissues such as muscle and nerves has been well-established (25, 26, 27, 28), but its function in early embryonic development remains unclear. Interestingly, studies have shown that creatine and its key metabolic enzymes may be involved in the regulation of cell proliferation and differentiation (29, 30, 31, 32). Our previous ultrasensitive metabolomic analysis revealed that creatine relative abundance gradually increased during oocyte maturation in sheep, whereas it progressively decreased from the 8-cell embryo to the blastocyst stage (33). This dynamic change suggests that creatine may play a crucial role in early embryonic development.

These findings have led us to propose a scientific hypothesis: creatine metabolism may be activated during the period from the 8-cell to the blastocyst stage. By regulating cellular energy states, it influences downstream cytoskeletal dynamics and key signaling pathways such as Hippo, precisely regulating lineage differentiation and blastocyst formation. However, this hypothesis still lacks direct experimental evidence and mechanistic explanation. In particular, the core molecular connection points between creatine metabolism and the known regulatory networks for lineage differentiation remain unclear. This study aims to systematically elucidate these issues.

Therefore, through the integration of *in vivo* and *in vitro* loss-of-function and rescue experiments, transcriptome and translatome sequencing (T&T-seq), and molecular cell biology approaches, this study reveals that creatine metabolism functions as an upstream metabolic hub that supports a regulatory axis of "energy buffering–F-actin assembly–YAP nuclear localization" to precisely control TE formation and blastocyst development in early mouse embryos. This work not only fills a critical gap in the field of metabolic regulation during embryonic development but also provides new perspectives and a theoretical foundation for understanding the underlying mechanisms of metabolism-related reproductive disorders.

## Results

### Creatine metabolism is essential for mouse development from the 8-cell embryo to the blastocyst stage

**Figure S1:**
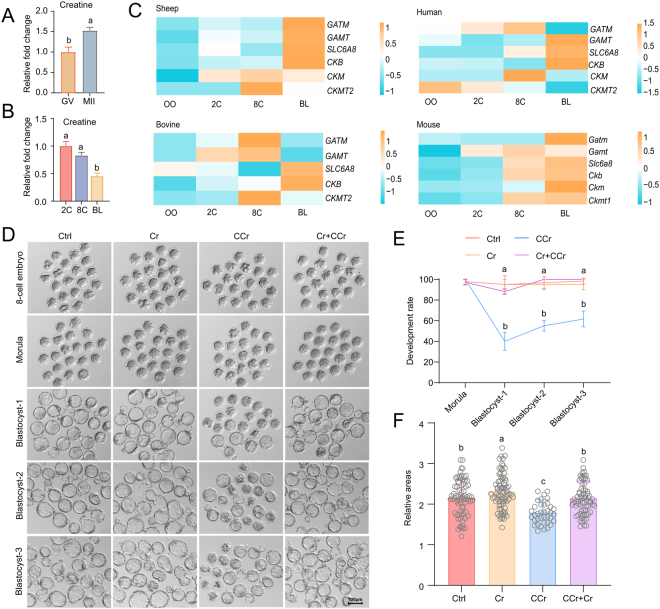


Our previous integrated analysis of ultrasensitive metabolomics and single-cell transcriptomics revealed creatine as a key missing metabolite in the ovine embryo *in vitro* culture system (33). Creatine relative abundance gradually increases during sheep oocyte maturation but significantly decreases from the 8-cell embryo to the blastocyst stage (Fig. S1, *A* and *B*), suggesting potential utilization of creatine by the embryos. Furthermore, analysis of multi-species transcriptome data (33, 34, 35, 36) revealed high expression levels of creatine transporter (*Slc6a8)* and creatine kinase (*Ckb)* during the blastocyst stage (Fig. S1*C*), suggesting that the creatine metabolism system plays a critical role in blastocyst formation.

**Figure 1:**
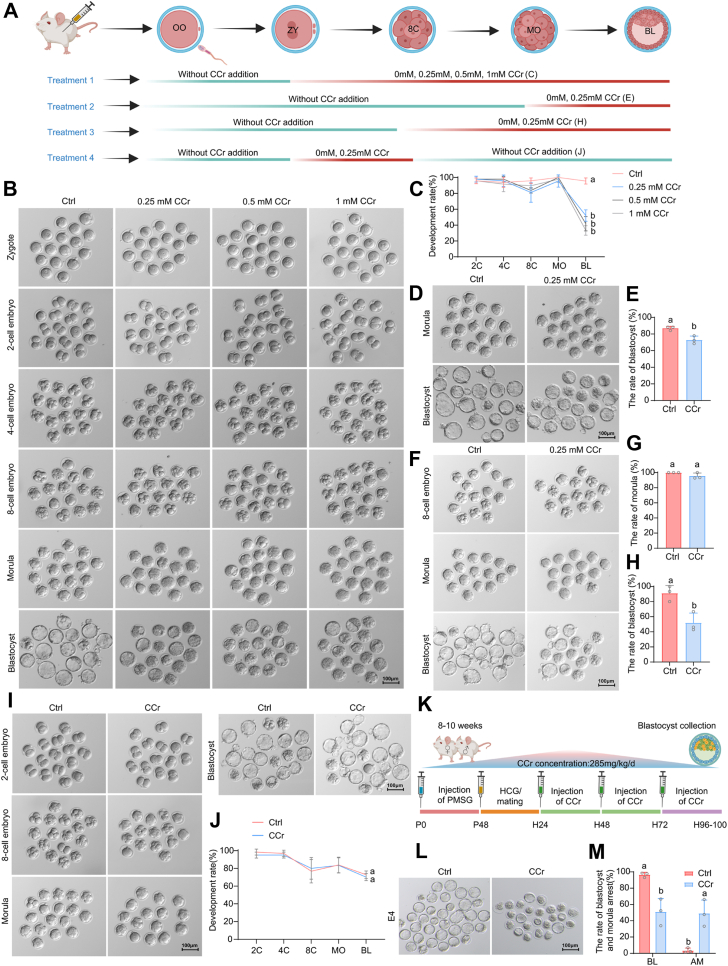
**Creatine metabolism is essential for mouse development from the 8-cell embryo to blastocyst stage.***A*, schematic diagram of CCr intervention at different developmental stages. *B*, representative images of embryos from zygote to blastocyst stage in control and CCr-treated groups (0.25, 0.5, 1 mM). Scale bar: 100 μm. *C*, embryo development rates for each group in *B*. Control (n = 48), 0.25 mM CCr (n = 54), 0.5 mM CCr (n = 54), and 1 mM CCr (n = 58). *D*, Representative images of morulae and blastocysts from control and CCr-treated groups when treated only from morula to blastocyst stage. Scale bar: 100 μm. *E*, blastocyst rate after treatment during this stage. Control (n = 53), CCr (n = 55). *F*, representative images of 8-cell embryos, morulae, and blastocysts when treated only from 8-cell to blastocyst stage. Scale bar: 100 μm. *G*, morula rate after treatment during this stage. *H*, blastocyst rate after treatment during this stage. Control (n = 45), CCr (n = 44). *I*, representative images of embryos at each stage from control and CCr (0.25 mM)-treated groups when treated only from zygote to 8-cell stage. Scale bar: 100 μm. *J*, Embryo development rate after treatment during this stage. *K*, Schematic diagram of *in vivo* CCr intervention. *L*, representative images of blastocysts from control and CCr-treated groups *in vivo*. Scale bar: 100 μm. *M*, Blastocyst rate and morula arrest rate in control and CCr-treated groups *in vivo*. Control (n = 269), CCr (n = 259). Error bars represent mean ± SD. Different letters (a, b, c, d) indicate statistically significant differences between groups (*p* < 0.05), exact *p*-values are provided in Table S2. Data were analyzed by one-way ANOVA followed by Tukey’s multiple comparison test for (*C*), and unpaired two-tailed *t* test for (*E, G, H, J, M*). *N* = 3 independent experiments for (*C, E, G, H, M*); *N* = 4 independent experiments for (*J*). Capital “N” indicates the number of biological replicates, and lowercase “n” indicates the number of embryos. AM, arrested morula; BL, blastocyst; E4, day four *in vivo* embryos.

To validate this hypothesis, we treated embryos at different stages with the creatine metabolism inhibitor cyclocreatine (CCr) (Fig. 1*A*). Zygotes were continuously exposed to varying concentrations (0, 0.25, 0.5, and 1 mM) of CCr until the blastocyst stage (full-stage). The results showed that CCr treatment had no significant effect on cleavage, 4-cell, 8-cell, or morula rates. However, its dose-dependently inhibited blastocyst formation (Fig. 1, *B* and *C*) and induced developmental arrest at the morula stage. Based on dose-response analysis, 0.25 mM CCr was selected for subsequent experiments. This concentration significantly reduced the blastocyst rate (by 43.51%) without causing nonspecific toxicity.

Since full-stage treatment only affected blastocyst development, we hypothesized that creatine metabolism primarily functions during the morula-to-blastocyst transition. However, when CCr was applied only during this specific stage, the blastocyst rate decreased by only 14.08%, which was significantly lower than the 43.51% reduction observed with full-stage treatment (Fig. 1, *D* and *E*). Subsequently, the treatment phase was advanced to the 8-cell stage. The results showed that CCr treatment had no significant effect on the morula formation rate (Fig. 1, *F* and *G*), and its inhibitory effect on blastocyst formation was comparable to that of full-stage treatment (blastocyst rate decreased by 39.22% *versus* 43.51%) (Fig. 1, *F* and *H*). These results suggest that the regulatory role of creatine metabolism begins to accumulate from the 8-cell stage, and its primary functional window is concentrated during the morula-to-blastocyst transition. Furthermore, treatment from the zygote to the 8-cell stage had no impact on development at any stage (Fig. 1, *I* and *J*), confirming that creatine metabolism regulation begins at the 8-cell stage. An *in vivo* model also confirmed that blastocyst formation was significantly reduced in the CCr group, with 49.05% of embryos arrested at the morula stage (Fig. 1, *K*–*M*). Together, these findings provide strong evidence for a critical role of creatine metabolism in embryonic development.

### Creatine metabolism specifically regulates TE formation

**Figure 2:**
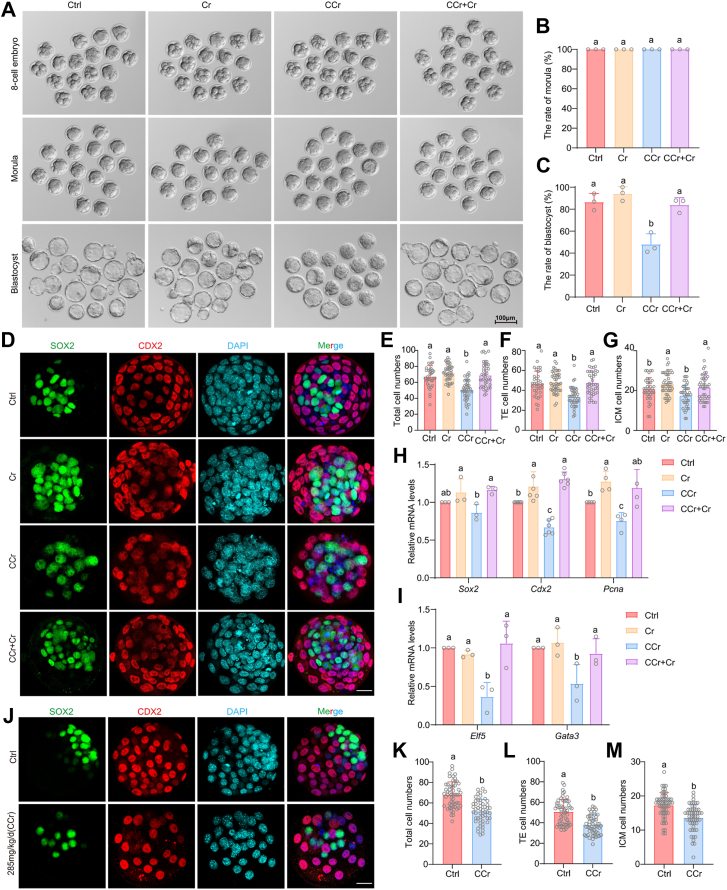
**Creatine metabolism specifically regulates TE formation.***A*, representative images of 8-cell embryos, morulae, and blastocysts in control, Cr, CCr, and rescue (CCr + Cr) groups. Scale bar: 100 μm. *B*, morula rates in each group. *C*, Blastocyst rates in each group. Control (n = 53), Cr (n = 51), CCr (n = 52), rescue (n = 49). *D*, Immunofluorescence staining images of SOX2, CDX2, and DAPI in blastocysts from each group. Scale bar: 50 μm. *E*-*G*, total, TE, and ICM cell numbers in blastocysts from each group. Control (n = 37), Cr (n = 46), CCr (n = 43), rescue (n = 43). *H*, relative mRNA expression levels of *Sox2*, *Cdx2*, and *Pcna* in blastocysts. *I*, relative mRNA expression levels of *Elf5* and *Gata3* in blastocysts. *J*, immunofluorescence staining images of SOX2, CDX2, and DAPI in blastocysts from control and CCr-treated groups *in vivo*. Scale bar: 50 μm. *K*–*M*, total, TE, and ICM cell numbers in blastocysts. Control (n = 56), CCr (n = 53). Error bars represent mean ± SD. Different letters (a, b, c, d) indicate statistically significant differences between groups (*p* < 0.05), exact *p*-values are provided in Table S2. Data were analyzed by one-way ANOVA followed by Tukey’s multiple comparison test for (*B, C, E, F, G, H, I*), and unpaired two-tailed *t* test for (*K, L, M*). *N* = 3 independent experiments for (*B, C, E, F, G, I*). For (*H*): N = 3 for *Sox2*, N = 4 for *Pcna*, N = 6 for *Cdx2*. CDX2, caudal type homeobox 2; SOX2, SRY-box transcription factor 2. ICM, inner cell mass; TE, trophectoderm.

To clarify the role of creatine during the 8-cell embryo to blastocyst stage and exclude nonspecific toxicity of CCr, we conducted creatine rescue experiments. 8-cell embryos were divided into four groups: control, creatine group (Cr, 1 mM), CCr group (0.25 mM), and rescue group (CCr + Cr). None of the treatments affected the morula rate (Fig. 2, *A* and *B*). Creatine supplementation alone did not influence the blastocyst rate (94.08 ± 3.62% *versus* 87.10 ± 4.42%, *p* > 0.05), whereas CCr significantly reduced the blastocyst rate (48.15 ± 5.42% *versus* 87.10 ± 4.42%, *p* < 0.05). This effect was restored to control levels by co-supplementation with creatine (87.99 ± 6.80% *versus* 87.10 ± 4.42%, *p* > 0.05) (Fig. 2, *A* and *C*). These findings indicate that creatine deficiency is a key cause of impaired blastocyst formation. Furthermore, extending the duration of *in vitro* culture revealed that approximately 22% of CCr-treated embryos exhibited delayed blastocyst formation, while about 37% remained developmentally arrested (Fig. S1, *D* and *E*), with reduced blastocyst area (Fig. S1*F*). These results further demonstrate that inhibition of creatine metabolism not only delays blastocyst formation but, more importantly, leads to embryonic developmental arrest.

Given that morula arrest coincides spatiotemporally with the first lineage differentiation, we hypothesized that creatine metabolism is involved in regulating cell fate determination. We utilized SOX2 and CDX2 staining to distinguish between the ICM and the TE within blastocysts. Compared with the control group, CCr-treated blastocysts exhibited a significant reduction in total and TE cell numbers (Fig. 2, *D*–*F*), whereas the ICM cell numbers showed no significant difference (Fig. 2, *D* and *G*). Exogenous creatine supplementation completely restored both total and TE cell numbers (Fig. 2, *E* and *F*), indicating that creatine metabolism may specifically regulate TE formation. RT-qPCR results revealed that, compared to the control group, the expression of the TE marker gene *Cdx2* and the proliferation gene *Pcna* was downregulated in CCr-treated blastocysts, and this effect could be rescued by creatine supplementation (Fig. 2*H*). In contrast, the ICM marker gene *Sox2* remained unchanged (Fig. 2*H*). Other key TE genes, including *Elf5* and *Gata3*, exhibited a similar trend (Fig. 2*I*), further supporting the specific regulatory role of creatine metabolism in TE formation. *In vivo* models likewise confirmed that CCr treatment significantly reduced total, TE, and ICM cell numbers in blastocysts (Fig. 2, *J*–*L*). These findings highlight the important role of creatine metabolism in trophectoderm formation during early mouse embryogenesis.

### Creatine metabolism promotes TE formation by regulating YAP nuclear localization

**Figure 3:**
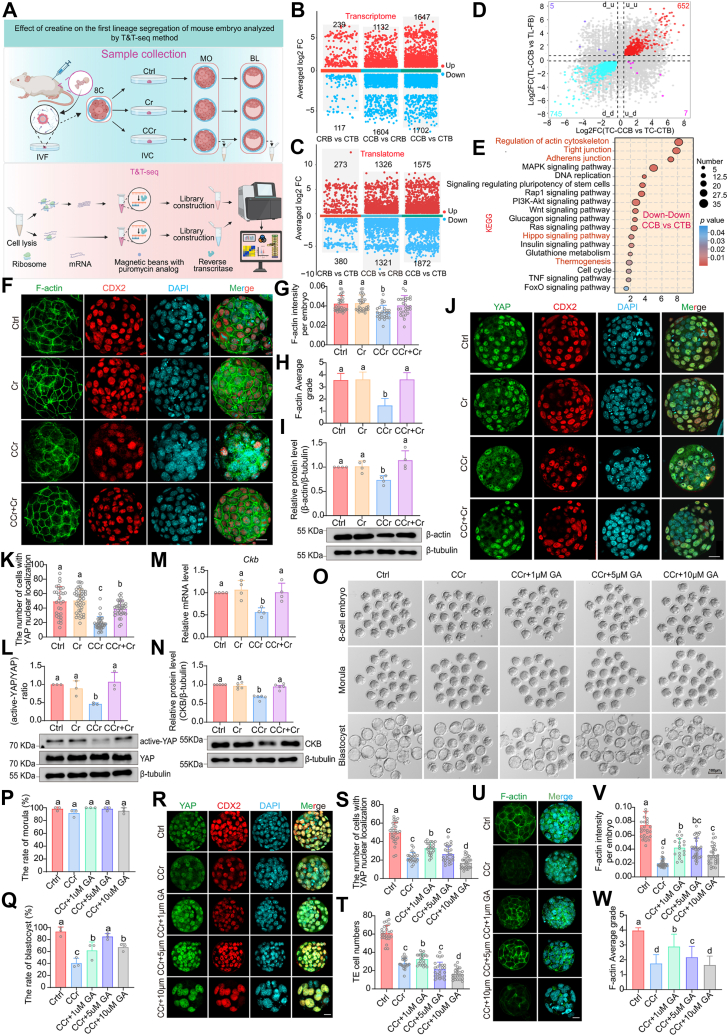
**Creatine metabolism promotes TE formation by regulating YAP nuclear localization.***A*, flowchart for sample collection and transcriptome/translatome sequencing analysis. *B*–*C*, Volcano plots showing differentially expressed genes in the transcriptome (*B*) and translatome (*C*) between groups at the blastocyst stage. *D*, nine-quadrant diagram displaying genes commonly up- and down-regulated in both transcriptome and translatome in the CCr group compared to control. *E*, KEGG pathway enrichment analysis was performed on the downregulated genes from (*D*). *F*, immunofluorescence staining images of F-actin, CDX2, and DAPI in blastocysts from each group. Scale bar: 50 μm. *G*, mean fluorescence intensity of F-actin in blastocysts from each group. *H*, average polymerization grade of F-actin in blastocysts from each group (grading criteria shown in Fig. S3*A*). Control (n = 33), Cr (n = 31), CCr (n = 26), and rescue (n = 29). *I*, β-actin Western blot representative bands and relative gray values in blastocysts from each group. *J*, immunofluorescence staining images of YAP, CDX2, and DAPI in blastocysts from each group. Scale bar: 50 μm. *K*, number of cells with YAP nuclear localization in blastocysts from each group. Control (n = 31), Cr (n = 41), CCr (n = 34), and rescue (n = 34). *L*, active-YAP and total YAP proteins Western blot representative bands and relative gray values in blastocysts from each group. *M*, The relative mRNA expression level of the *Ckb* gene in blastocysts from each group. *N*, CKB protein Western blot representative bands and relative *gray* values in blastocysts from each group. *O*, representative images of embryos at each stage from control, CCr, and CCr combined with different concentrations of YAP agonist GA (1, 5, 10 μM) groups. Scale bar: 100 μm. *P*, morula rates in each group. *Q*, Blastocyst rates in each group. Control (n = 60), CCr (n = 59), 1 μM GA (n = 61), 5 μM GA (n = 60), and 10 μM GA (n = 61). *R*, immunofluorescence staining images of YAP, CDX2, and DAPI in blastocysts from each group. Scale bar: 50 μm. *S*, the number of cells with YAP nuclear localization in blastocysts from each group. *T*, TE cell numbers in blastocysts from each group. Control (n = 28), CCr (n = 24), 1 μM GA (n = 24), 5 μM GA (n = 27), and 10 μM GA (n = 26). *U*, immunofluorescence staining images of F-actin in blastocysts from each group. Scale bar: 50 μm. *V*, the mean fluorescence intensity of F-actin in blastocysts from each group. *W*, the mean polymerization grade of F-actin in blastocysts from each group. Control (n = 29), CCr (n = 35), 1 μM GA (n = 18), 5 μM GA (n = 32), and 10 μM GA (n = 31). Error bars represent mean ± SD. Different letters (a, b, c, d) indicate statistically significant differences between groups (*p* < 0.05), exact *p*-values are provided in Table S2. Data were analyzed by one-way ANOVA followed by Tukey’s multiple comparison test. *N* = 3 independent experiments for (*G, H, I, L, P, Q, S, T, V, W*); *N* = 4 independent experiments for (*K, M*); *N* = 5 independent experiments for (*N*). YAP, Yes-associated protein; F-actin, filamentous actin; CTB, control group blastocyst; CRB, creatine group blastocyst; CCB, cyclocreatine group blastocyst.

**Figure S2:**
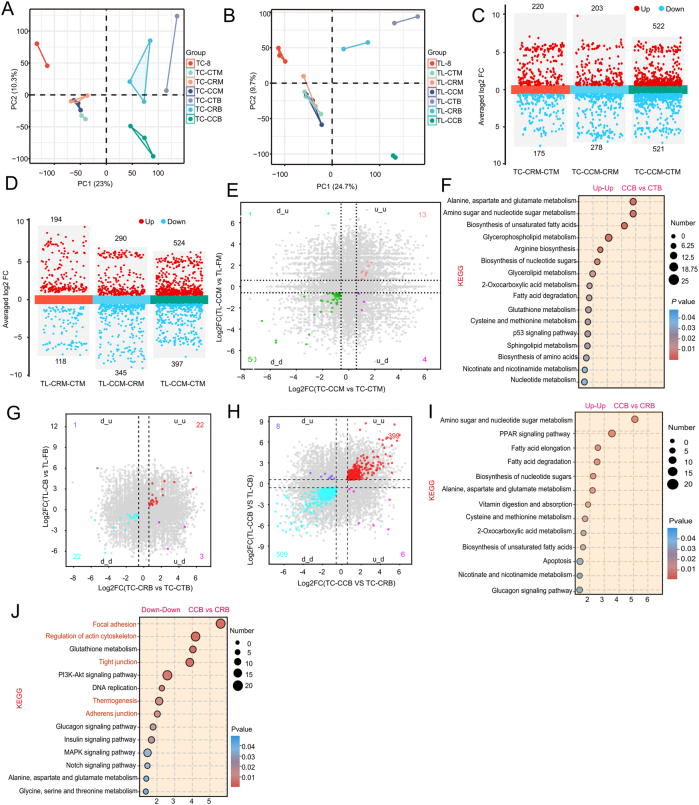


To further investigate the underlying mechanisms, we performed transcriptome and translatome sequencing on embryos under different treatments at the morula and blastocyst stages (Fig. 3*A*). Principal component analysis (PCA) showed minimal differences between groups at the morula stage, whereas significant divergence emerged at the blastocyst stage (Fig. S2, *A* and *B*). At the morula stage, compared with the control group, the CCr group exhibited 522 upregulated genes and 521 downregulated genes at the transcriptional level (Figs S2*C*), and 524 genes were upregulated and 397 genes were downregulated at the translational level (Fig. S2*D*). Nine-quadrant diagram analysis revealed few genes with coordinated upregulation or downregulation between the transcriptome and translatome (Fig. S2*E*), indicating poor transcript-translation coupling. In contrast, at the blastocyst stage, compared with control group, the CCr group showed 1647 upregulated and 1702 downregulated genes at the transcriptional level (Figs. 3*B*), and 1575 upregulated and 1872 downregulated genes at the translational level (Fig. 3*C*). Nine-quadrant diagram analysis identified 652 genes with coordinated upregulation and 745 genes with coordinated downregulation (Fig. 3*D*). KEGG enrichment analysis demonstrated that upregulated genes were primarily enriched in pathways related to amino acid metabolism, amino and nucleotide sugar metabolism, and lipid metabolism (Fig. S2*F*), whereas downregulated genes were enriched in pathways such as actin cytoskeleton regulation, tight junctions, adherens junctions, and the Hippo signaling pathway (Fig. 3*E*). Furthermore, 22 genes exhibited coordinated upregulation or downregulation between the Cr group and the control group (Fig. S2*G*). Between the CCr group and the Cr group, 399 genes showed coordinated upregulation and 509 genes showed coordinated downregulation (Fig. S2*H*), with the enriched pathways presented in Fig. S2, *I* and *J*.

**Figure S3:**
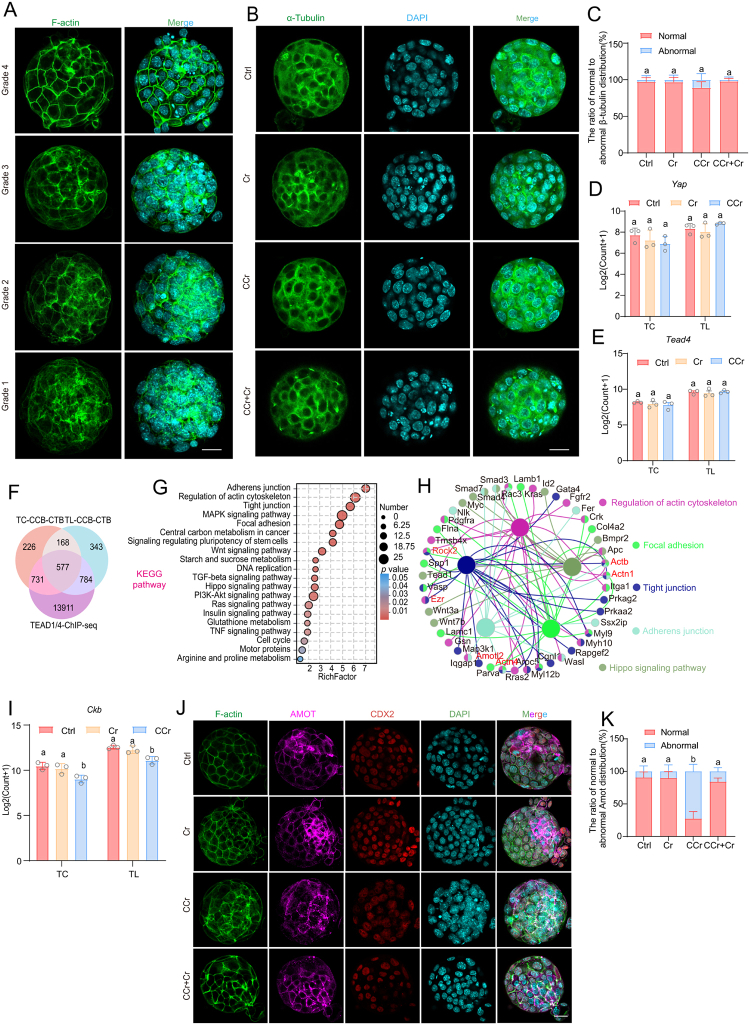


Given the central roles of the actin cytoskeleton and the Hippo pathway in TE differentiation, we examined the expression and distribution of F-actin in blastocysts. Immunofluorescence and Western Blot results showed that inhibiting creatine metabolism significantly reduced F-actin fluorescence intensity (Fig. 3, *F* and *G*), its polymerization grade (Fig. 3*H*) (evaluation criteria for polymerization grades are shown in Fig. S3*A*), and the abundance of β-actin (the precursor of F-actin formation) (Fig. 3*I*). Since extensive interactions exist among cytoskeletal networks (37, 38), to assess the specificity of creatine metabolism's role, we further examined whether the microtubule network was similarly affected. Immunofluorescence staining revealed that the α-tubulin network remained intact in CCr-treated blastocysts, with distribution patterns similar to those in the control group (Fig. S3, *B* and *C*). Collectively, these results confirm that inhibiting creatine metabolism specifically affects F-actin without causing global cytoskeletal disruption. It is well established that F-actin regulates the nuclear localization of the Hippo effector YAP. YAP translocates into the nucleus and cooperates with TEADs to activate the transcription of TE-related genes (39). Sequencing results indicated that inhibiting creatine metabolism did not alter the transcriptional or translational levels of *Yap* and *Tead4* (Fig. S3, *D* and *E*). However, the experiment revealed a significant decrease in the number of cells with nuclear YAP localization (Fig. 3, *J* and *K*), and the active (non-phospho)-YAP/YAP ratio decreased markedly (Fig. 3*L*). YAP must bind to TEADs to regulate gene transcription (10, 14). Intersecting the downregulated differentially expressed genes in the CCr group with TEAD1/4 ChIP-seq data (40) yielded 577 overlapping genes (Fig. S3*F*). These genes were also enriched in KEGG pathways such as adherens junctions, actin cytoskeleton regulation, and gap junctions (Fig. S3*G*). Gene network diagrams of these pathways are shown in Fig. S3*H*, suggesting that inhibiting creatine metabolism may disrupt actin cytoskeleton homeostasis by impairing YAP-TEADs function. Interestingly, CKB was also among these 577 overlapping genes. After CCr treatment, its transcriptional and translational levels (Fig. S3*I*), as well as its mRNA and protein levels, were significantly reduced (Fig. 3, *M* and *N*).

Further investigation revealed that inhibiting creatine metabolism led to abnormal distribution of AMOT (angiomotin), a key protein regulating YAP nuclear localization (Fig. S3, *J* and *K*). To determine whether impaired YAP nuclear localization is a primary cause of embryonic developmental arrest, F-actin abnormalities, and TE reduction, we supplemented the CCr-treated group with GA-017, an agonist that promotes YAP nuclear localization. The results showed no significant differences in morula rates among the treatment groups (Fig. 3, *O* and *P*). However, 5 μM GA completely restored the blastocyst rate (Fig. 3, *O* and *Q*), whereas only 1 μM GA partially rescued YAP nuclear localization (Fig. 3, *R* and *S*), TE cell numbers (Fig. 3, *R* and *T*), and F-actin fluorescence intensity and polymerization grade (Fig. 3, *U*–*W*). These findings indicate that creatine metabolism can influence F-actin homeostasis and TE formation by regulating YAP nuclear localization, yet the underlying regulatory network is likely more complex.

### Inhibition of creatine metabolism impedes YAP nuclear localization potentially through activating AMPK, thereby leading to abnormal glycolysis in blastocysts

**Figure 4:**
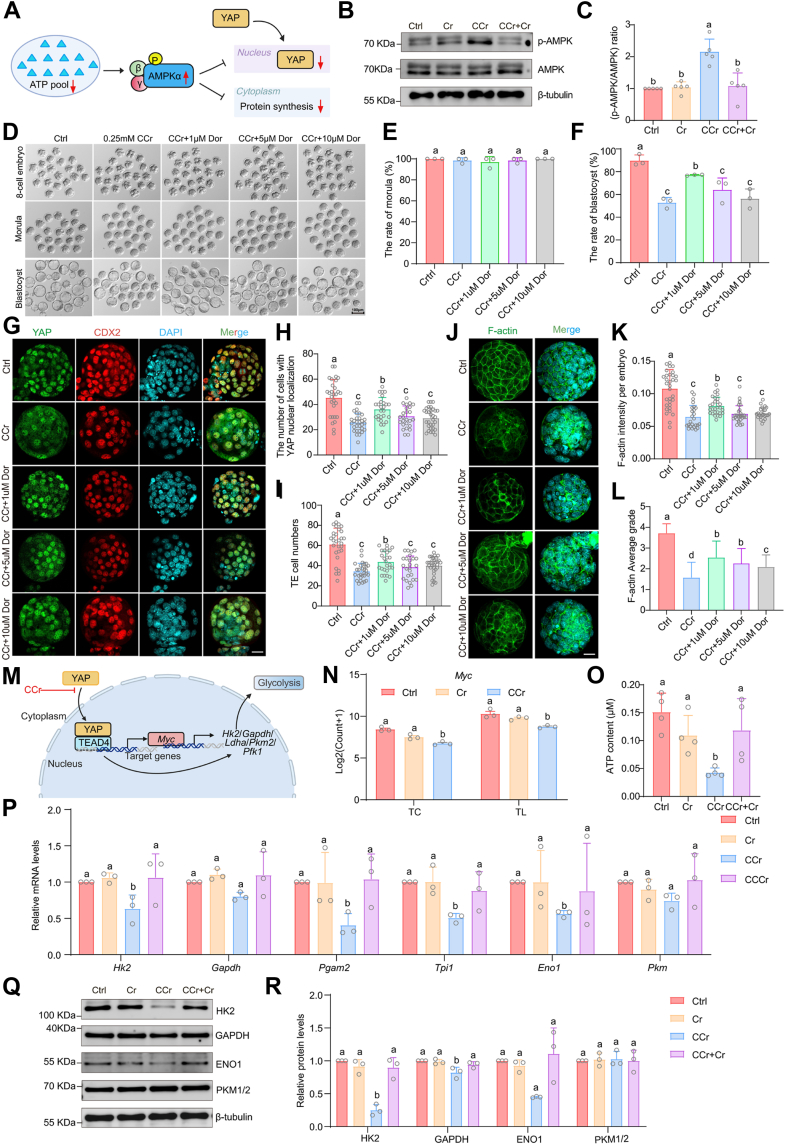
**Inhibition of creatine metabolism impedes YAP nuclear localization potentially through activating AMPK, thereby leading to abnormal glycolysis in blastocysts.***A*, schematic diagram illustrating how ATP reduction activates p-AMPK. *B*, Representative Western blot bands of AMPK and p-AMPK proteins in blastocysts from each group. *C*, relative ratio of p-AMPK/AMPK in blastocysts from each group. *D*, representative images of embryos at each stage from control, CCr, and CCr combined with different concentrations of AMPK inhibitor Dor (1, 5, 10 μM) groups. Scale bar: 100 μm. *E*, morula rates for each group. *F*, blastocyst rates for each group. Control (n = 60), CCr (n = 59), 1 μM Dor (n = 61), 5 μM Dor (n = 63), and 10 μM Dor (n = 61). *G*, immunofluorescence staining images of YAP, CDX2, and DAPI in blastocysts from each group. Scale bar: 50 μm. *H*, number of cells with YAP nuclear localization in blastocysts from each group. *I*, TE cell numbers in blastocysts from each group. Control (n = 28), CCr (n = 30), 1 μM Dor (n = 26), 5 μM Dor (n = 25), and 10 μM Dor (n = 31). *J*, Immunofluorescence staining images of F-actin in blastocysts from each group. Scale bar: 50 μm. *K*, mean fluorescence intensity of F-actin in blastocysts from each group. *L*, mean polymerization grade of F-actin in blastocysts from each group. Control (n = 33), CCr (n = 25), 1 μM Dor (n = 28), 5 μM Dor (n = 29), and 10 μM Dor (n = 24). *M*, Schematic diagram illustrating the impact of YAP dysfunction on glycolysis. *N*, transcriptome and translatome counts for the *Myc* gene in blastocysts from each group. *O*, Relative ATP content in blastocysts from each group. *P*, relative mRNA expression levels of glycolysis-related genes in blastocysts from each group. *Q*, Representative Western blot bands of HK2, GAPDH, ENO1, and PKM proteins in blastocysts from each group. *R*, analysis of relative grayscale values of the above glycolysis-related proteins in blastocysts from each group. Error bars represent mean ± SD. Different letters (a, b, c, d) indicate statistically significant differences between groups (*p* < 0.05). Exact *p*-values are provided in Table S2. Data were analyzed by one-way ANOVA followed by Tukey’s multiple comparison test. *N* = 3 independent experiments for (*E, F, H, I, K, L, P, R*); *N* = 4 independent experiments for (*C, O*). Upon special explanation: The representative image of the CCr + 10 μM Dor group in Figure 4J is the same as the Grade 2 reference image used for F-actin grading in Fig. S3*A*. During the F-actin grading assessment, we selected universal representative images to define each F-actin grade, which were not limited to any specific experimental treatment group. All F-actin grading results presented in Figure 4J were evaluated strictly based on the grading criteria established in Fig. S3*A*. We provide this explicit statement to avoid unnecessary misunderstanding of image reuse.

**Figure S4:**
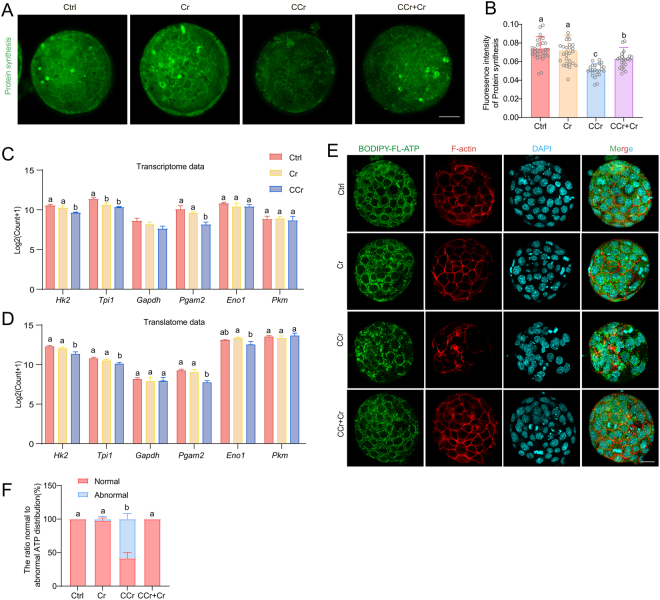


Creatine metabolism plays a crucial role in maintaining cellular energy homeostasis. To elucidate the potential mechanism by which it regulates YAP nuclear localization, we examined the expression level of the energy sensor p-AMPK in blastocysts, which can directly or indirectly regulate YAP phosphorylation (Fig. 4*A*). The results showed that inhibition of creatine metabolism significantly increased p-AMPK/AMPK ratio in blastocysts (Fig. 4, *B* and *C*) and reduced nascent protein synthesis (HPG signal) (Fig. S4, *A* and *B*). To investigate whether p-AMPK activation contributes to developmental arrest and impaired YAP nuclear localization, we added the p-AMPK inhibitor dorsomorphin (Dor) to the CCr group. The results revealed no significant differences in morula rates among the treatment groups (Fig. 4, *D* and *E*). Notably, 1 μM Dor partially rescued blastocyst development rates (Fig. 4, *D* and *F*) and significantly restored YAP nuclear localization (Fig. 4, *G* and *H*), TE cell numbers (Fig. 4, *G* and *I*), as well as F-actin fluorescence intensity and polymerization grades (Fig. 4, *J*–*L*). However, these indicators were not completely restored to control levels, suggesting that energy stress-induced elevation of p-AMPK is a partial contributor to YAP cytoplasmic retention and F-actin abnormalities.

YAP can directly or indirectly (by activating *Myc* transcription) regulate glycolysis (41, 42, 43, 44, 45) (Fig. 4*M*), a process closely associated with TE differentiation (44, 46). Sequencing data revealed that inhibition of creatine metabolism led to significant downregulation of *Myc* (Fig. 4*N*) as well as several glycolytic genes (*Hk2, Tpi1, Pgam2*) at both transcriptional and translational levels (Fig. S4, *C* and *D*). Experimental validation confirmed that in the CCr group, blastocysts exhibited reduced ATP content (Fig. 4*O*) and abnormal ATP distribution (Fig. S4, *E* and *F*), the levels of glycolytic genes (*Hk2*, *Pgam2*, *Tpi1*, *Eno1*) and proteins (HK2, GAPDH, and ENO1) were significantly reduced. These changes were reversed by exogenous creatine supplementation (Fig. 4, *P*–*R*). These findings suggest that inhibition of creatine metabolism may disrupt blastocyst glycolysis, potentially through suppressing YAP nuclear localization.

### Inhibition of creatine metabolism reduces ATP levels, which impairs F-actin assembly and consequently hinders YAP nuclear localization

**Figure 5:**
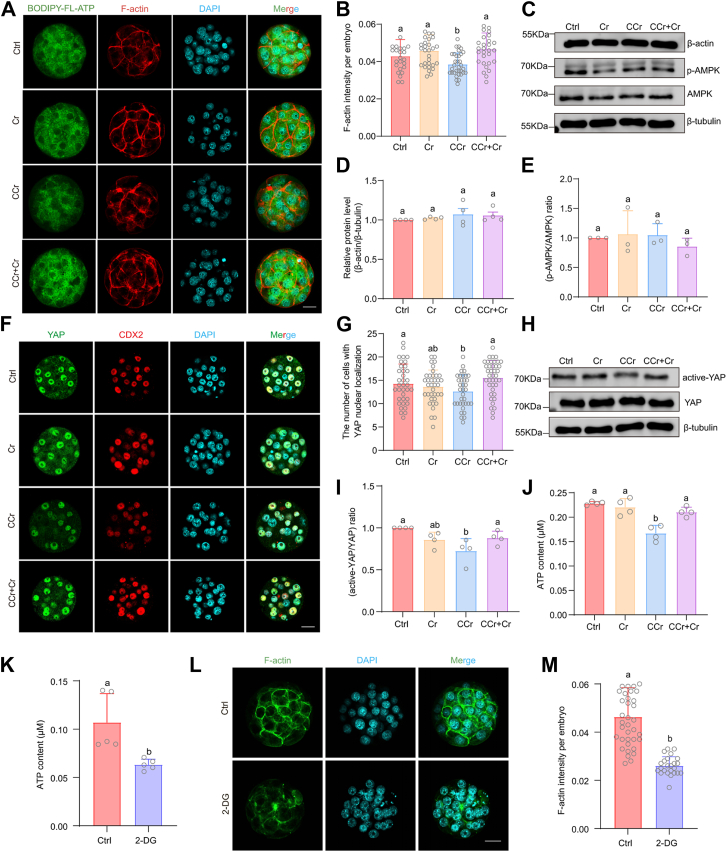
**Inhibition of creatine metabolism impairs F-actin assembly and blocks YAP nuclear localization by reducing ATP levels.***A*, immunofluorescence staining images of F-actin, ATP (labeled with a fluorescent probe), and DAPI in morulae from each group. Scale bar: 50 μm. *B*, mean fluorescence intensity of F-actin in morulae from each group. Control (n = 25), Cr (n = 30), CCr (n = 33), and rescue (n = 30). *C*, representative Western blot bands of β-actin, p-AMPK, and AMPK proteins in morulae from each group. *D*-*E*, Relative grayscale values of β-actin protein and the p-AMPK/AMPK ratio in morulae from each group, respectively. *F*, immunofluorescence staining images of YAP, CDX2, and DAPI in morulae from each group. Scale bar: 50 μm. *G*, number of cells with YAP nuclear localization in morulae from each group. Control (n = 37), Cr (n = 37), CCr (n = 35), and rescue (n = 40). *H*, representative Western blot bands of active-YAP and YAP proteins in morulae from each group. *I*, active-YAP/YAP ratio in morulae from each group. *J*, ATP content in morulae from each group. *K*, Relative ATP content in morulae from the control and the 2-deoxy-D-glucose (2-DG, a glycolysis inhibitor) treatment group. *L*, Immunofluorescence staining images of F-actin and DAPI in morulae from each group. Scale bar: 50 μm. *M*, mean fluorescence intensity of F-actin in morulae from each group. Control (n = 38) and 2-DG treatment group (n = 24). Error bars represent mean ± SD. Different letters (a, b, c, d) indicate statistically significant differences between groups (*p* < 0.05), exact *p*-values are provided in Table S2. Data were analyzed by one-way ANOVA followed by Tukey’s multiple comparison test for (*B, D, G, I, J*), and unpaired two-tailed *t* test for (*K, M*). *N* = 3 independent experiments for (*B, E, G, M*); *N* = 4 independent experiments for (*D, I, J*); *N* = 6 independent experiments for (*K*).

**Figure S5:**
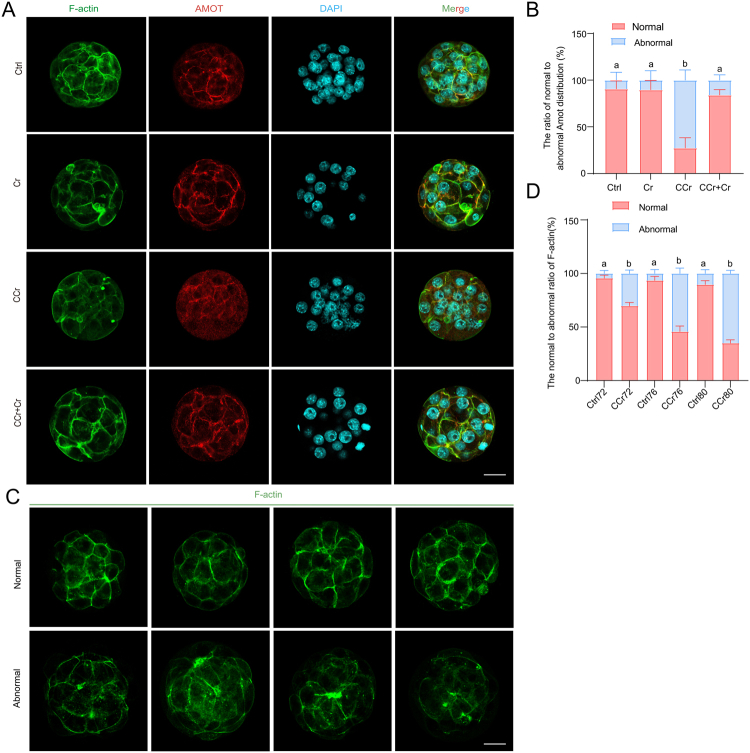


Both F-actin and AMPK can influence YAP nuclear localization. To clarify the temporal relationship among F-actin abnormalities, p-AMPK activation, and decreased YAP nuclear localization, we examined phenotypes at the morula stage. The results showed that CCr treatment at the morula stage had already reduced F-actin fluorescence intensity (Fig. 5, *A* and *B*) and disrupted AMOT distribution (Fig. S5, *A* and *B*). Furthermore, F-actin polymerization abnormalities intensify as development progresses (Fig. S5, *C* and *D*). At this stage, no significant differences were observed between the CCr group and the control group in β-actin protein levels (Fig. 5, *C* and *D*) and the p-AMPK/AMPK ratio (Fig. 5, *C* and *E*). However, the number of cells with nuclear YAP localization was significantly reduced (Fig. 5, *F* and *G*), and the active-YAP/YAP ratio had decreased markedly (Fig. 5, *H* and *I*). These findings indicate that inhibition of creatine metabolism primarily impairs F-actin assembly, which subsequently hinders YAP nuclear localization.

To determine whether the F-actin abnormalities in morulae induced by inhibition of creatine metabolism are caused by ATP deficiency, we measured ATP levels in morulae. The results showed that ATP content was significantly reduced in the CCr-treated group (Fig. 5*J*). To further confirm whether ATP deficiency directly leads to F-actin abnormalities, we added the glycolysis inhibitor 2-DG to reduce ATP levels. The results demonstrated that 2-DG treatment reduced ATP content (Fig. 5*K*) and weakened F-actin fluorescence intensity while disrupting its assembly (Fig. 5, *L* and *M*). This indicates that the impairment of F-actin assembly caused by inhibition of creatine metabolism can be attributed, at least in part, to insufficient energy supply.

### CKB promotes F-actin assembly potentially through interaction with ARP2 and by harnessing the creatine-phosphocreatine system for energy supply

**Figure 6:**
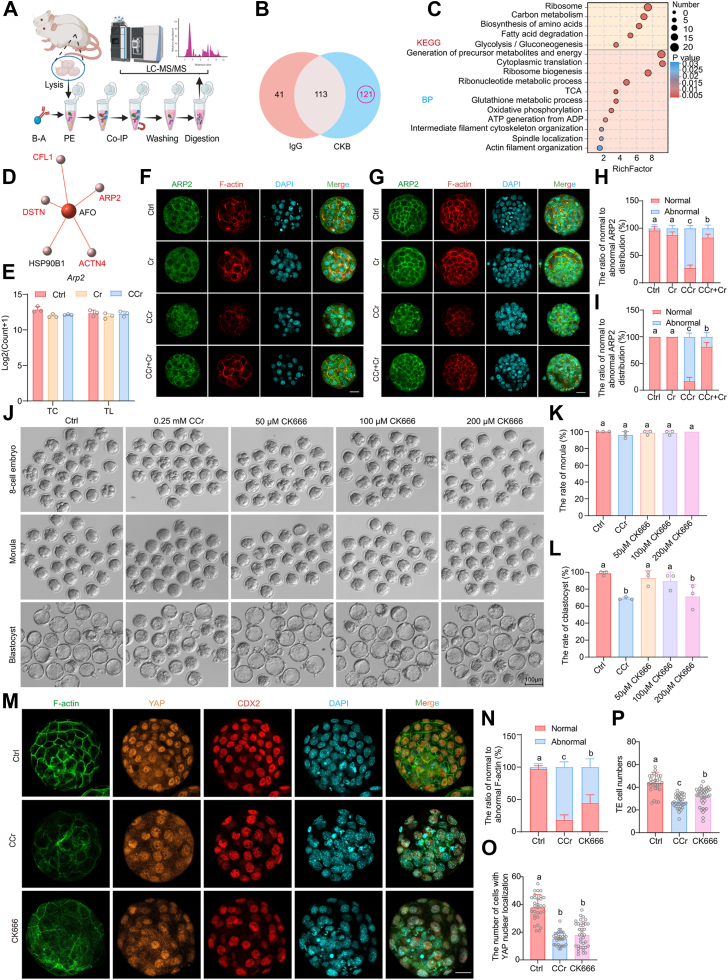
**CKB promotes F-actin assembly potentially through interaction with ARP2 and by harnessing the creatine-phosphocreatine system for energy supply.***A*, schematic diagram of the Co-IP-MS experimental procedure: using mouse ovary samples, CKB antibody was used for co-immunoprecipitation to screen for proteins interacting with CKB. *B*, Venn diagram of proteins interacting with CKB compared to IgG control. *C*, KEGG pathway and biological process (BP) enrichment analysis based on the 121 CKB-interacting proteins from (*B*). *D*, network diagram of proteins involved in actin filament assembly. *E*, transcriptome and translatome counts of *Arp2* genes in morulae from each group. *F*-*G*, immunofluorescence staining images of ARP2, F-actin, and DAPI in morulae and blastocysts from each group. Scale bar: 50 μm. *H*, proportion of morulae showing normal *versus* abnormal ARP2 distribution in each group. Control (n = 25), Cr (n = 33), CCr (n = 25), and rescue (n = 30). *I*, proportion of blastocysts with normal *versus* abnormal ARP2 localization in each group. Control (n = 30), Cr (n = 32), CCr (n = 23), and rescue (n = 28). *J*, representative images of embryos at various stages in the control group, CCr group, and groups treated with different concentrations of the ARP2 inhibitor CK666 (50, 100, 200 μM). Scale bar: 100 μm. *K*, morula rate in each group. *L*, Blastocyst rate in each group. Control group (n = 60), CCr group (n = 59), 50 μM CK666 (n = 61), 100 μM CK666 (n = 68), 200 μM CK666 (n = 73). *M*, immunofluorescence staining images of F-actin, YAP, CDX2, and DAPI in blastocysts from each group. Scale bar: 50 μm. *N*, proportion of normal *versus* abnormal F-actin in blastocysts in each group. *O*, number of cells with nuclear YAP localization in blastocysts in each group. *P*, TE cell numbers in blastocysts in each group. Control group (n = 30), CCr group (n = 33), CK666 group (n = 37). Error bars represent mean ± SD. Different letters (a, b, c, d) indicate statistically significant differences between groups (*p* < 0.05), exact *p*-values are provided in Table S2. Data were analyzed by one-way ANOVA followed by Tukey’s multiple comparison test. *N* = 3 independent experiments for (*H, I, K, L, N, O, P*).

To further elucidate the potential mechanisms by which creatine metabolism regulates F-actin assembly, we employed Co-IP-MS to screen for proteins interacting with CKB (Fig. 6*A*). A total of 121 candidate proteins were identified (Fig. 6*B*), primarily enriched in pathways related to metabolism, ribosome biogenesis, cytoplasmic translation, and cytoskeleton assembly (Fig. 6*C*). Relevant proteins involved in these pathways are shown in Fig. S6, *A*-*F*. Proteins participating in actin filament assembly included ARP2, ACTN4, DSTN, and CFL1 (Fig. 6*D*). ARP2 regulates actin filament branching and nucleation, ACTN4 crosslinks actin filaments to maintain structural stability, while DSTN and CFL1 collectively mediate actin depolymerization and remodeling to ensure dynamic equilibrium. Among these, ARP2-driven actin assembly is an ATP-dependent process (47). Transcriptome and translatome data indicated that inhibition of creatine metabolism did not affect the transcriptional or translational levels of *Arp2*, *Actn4*, *Dstn*, and *Cfl1* in morulae (Figs. 6*E* and S6, *G–I*). However, immunofluorescence analysis showed an abnormal ARP2 distribution in CCr-treated morulae: control embryos displayed even ARP2 distribution, clear cell boundaries, and regular morphology, whereas the treated group exhibited uneven distribution, blurred cell boundaries, and irregular morphology (Fig. 6, *F* and *H*). This abnormality persisted at the blastocyst stage. In the control group, ARP2 displayed a distinct grid-like pattern along cell borders and partially colocalized with F-actin, whereas in the CCr group, ARP2 signals were scattered, lacking a clear grid pattern, and cell boundaries remained blurred (Fig. 6, *G* and *I*). This likely occurs because inhibiting creatine metabolism reduces ATP levels, which impairs ARP2 activation and prevents its proper recruitment to the cytoskeleton, thereby hindering F-actin assembly.

To determine whether ARP2 inhibition phenocopies the effects induced by CCr treatment, we assessed embryonic development, F-actin assembly, YAP nuclear localization, and TE cell numbers following treatment with different concentrations of the ARP2 inhibitor CK666. Our results showed that, compared with the control group, 50 and 100 μM CK666 did not inhibit embryonic development (Fig. 6, *J–L*). Although 200 μM CK666 did not affect the formation rate of morulae, it significantly inhibited blastocyst formation (Fig. 6, *J–L*). Furthermore, 200 μM CK666 led to abnormal ARP2 distribution (Fig. S6, *J* and *K*), disrupted F-actin assembly (Fig. 6, *M* and *N*), and significantly reduced YAP nuclear localization and TE cell numbers (Fig. 6, *M*, *O*, and *P*). The abovementioned phenotypes are consistent with those induced by inhibition of creatine metabolism. Together, these results suggest that ARP2 distribution abnormalities caused by energy insufficiency are a primary trigger for the subsequent series of defects.

Given that N-WASP functions as a key upstream activator of the Arp2/3 complex, we next asked whether inhibiting creatine metabolism affects N-WASP distribution. Immunofluorescence staining revealed that in control embryos, N-WASP was enriched at cell–cell junctions and partially colocalized with F-actin. In contrast, CCr treatment led to a striking alteration in N-WASP distribution, manifesting as a diffuse distribution throughout the cytoplasm (Fig. S6*L*). Quantitatively, 83.3% of CCr-treated embryos exhibited abnormal N-WASP localization, compared with only 7.33% in the control group (Fig. S6*M*). Furthermore, co-treatment with CCr and creatine completely restored this abnormal distribution (Fig. S6*M*). These results indicate that creatine metabolism affects the subcellular localization of N-WASP, providing a potential mechanistic explanation for the downstream defects in Arp2/3 distribution, F-actin assembly, and trophectoderm formation.

## Discussion

This study is the first to systematically elucidate the central role of creatine metabolism in TE formation during early mouse embryonic development. We found that creatine metabolism is essential for the development of mouse embryos from the 8-cell embryo to the blastocyst stage. Disruption of this efficient creatine-phosphocreatine energy-buffering system specifically arrests embryos at the critical transition from morula to blastocyst and significantly impairs TE formation, while having no significant effect on the ICM. This observation suggests that differentiating TE precursor cells exhibit a particular dependence on the immediate, readily available ATP supply maintained by this system, likely to meet their intense energy demands during processes such as compaction, blastocoel formation, and rapid proliferation (48, 49, 50, 51).

At the molecular level, we reveal that creatine metabolism regulates TE formation through the axis of energy buffering–F-actin assembly–YAP nuclear localization. Inhibition of creatine metabolism reduces ATP levels and disrupts ARP2 distribution in morulae. ARP2, a subunit of the ARP2/3 complex, requires ATP binding to activate the complex and drive actin filament branching (47, 52, 53). Therefore, ATP depletion may impair ARP2 activation and recruitment, ultimately leading to aberrant F-actin assembly. Previous studies have shown that Arp3 deficiency specifically impairs TE function, resulting in blastocyst lethality without affecting the inner cell mass (54). In contrast, inhibition of the ARP2/3 complex with CK666 from the 8-cell stage leads to loss of embryo polarity and failure of both ICM and TE formation (55). In the present study, we inhibited ARP2/3 from the 8-cell stage to the blastocyst stage and found that partial embryos arrested at the morula stage, while those that successfully developed into blastocysts exhibited impaired F-actin assembly and reduced TE cell number, with normal ICM formation. This phenotypic discrepancy may be attributed to the fact that direct inhibition of the ARP2/3 complex completely abolishes F-actin assembly in all blastomeres, leading to global developmental failure. In contrast, inhibition of creatine metabolism only reduces ATP availability, thereby partially compromising ARP2/3 activation. TE formation requires exceptionally high levels of F-actin dynamics (56) and, therefore, may have a higher demand for ARP2/3 activity, rendering it more sensitive to energy stress, whereas ICM formation remains unaffected.

F-actin not only serves as the structural basis for maintaining cell morphology and polarity but also acts as a key upstream regulator of the Hippo signaling pathway. Studies have shown that the F-actin network, established and enriched at the apical membrane during compaction, inhibits LATS1/2 kinase activity, thereby preventing YAP phosphorylation and promoting its nuclear translocation (5, 57). Our results are highly consistent with this model: inhibition of creatine metabolism induces F-actin abnormalities, which directly cause a significant decrease in cells with YAP nuclear localization, thereby attenuating the ability of YAP to cooperate with the TEADs transcription complex in activating downstream critical genes of TE formation such as *Cdx2.* Furthermore, our findings indicate that inhibition of creatine metabolism does not significantly affect α-tubulin distribution. The underlying reason for this phenomenon may be that impaired creatine metabolism primarily leads to a reduction in intracellular ATP levels, whereas GTP serves as the critical energy substrate for microtubule assembly (58). Consequently, this inhibition has no substantial impact on microtubule assembly and distribution.

Given the downregulation of CKB expression upon creatine metabolism inhibition, we next asked whether CKB itself might be regulated by YAP-TEADs. Interestingly, CKB itself may be a transcriptional target of YAP-TEADs. This aligns with previous research in pancreatic cancer cells, which showed that interference with YAP leads to a significant downregulation of CKB protein levels (51). This suggests the potential existence of a positive feedback loop: initial creatine metabolic activity promotes YAP nuclear localization by ensuring energy supply, while activated YAP-TEADs complexes may further upregulate CKB expression. This loop could amplify and sustain the efficiency of the creatine metabolism system, thereby providing energy security for blastocoel expansion and TE formation. Such tight coupling between metabolism and transcriptional programs offers insights into the developmental robustness observed during embryogenesis.

Our study further demonstrates that the energy stress induced by inhibition of creatine metabolism exerts multi-layered effects. The decrease in ATP not only directly impairs cytoskeletal assembly but also activates the cellular energy sensor AMPK. Activated AMPK can directly or indirectly promote YAP phosphorylation (59), thereby amplifying developmental defects. The use of AMPK inhibitors partially rescued the reduction in blastocyst development rate caused by the inhibition of creatine metabolism, confirming that excessive AMPK activation is a significant contributing factor. This indicates that during energy-sensitive stages of embryonic development, preventing the triggering of energy stress signals is as crucial as maintaining an adequate supply of energy substrates. Previous studies have also revealed that energy stress in tumor cells promotes YAP inactivation through the AMPK–AMOTL1–LATS axis (60). Together, these findings indicate that AMPK activation induced by energy stress and its subsequent regulation of YAP signaling are conserved across different cell types.

Furthermore, our study links creatine metabolism to the glycolytic metabolism of the embryo. Previous research has established that YAP and its target gene *Myc* are key regulators of glycolysis (44, 50, 61, 62, 63, 64). The present study found that inhibition of creatine metabolism leads to a decrease in YAP function, accompanied by a downregulation of *Myc* and several key glycolysis-related genes. This suggests that creatine metabolism may indirectly shape embryonic glycolytic flux through YAP. As glycolysis serves as another major source of ATP, its attenuation could further exacerbate the cellular energy stress, potentially creating a vicious cycle and contributing to developmental arrest. This aligns with recent perspectives emphasizing the synergy and complementarity among metabolic pathways in supporting embryonic development (65, 66).

While this study identifies a core regulatory axis, several questions remain. First, direct biochemical evidence is required to confirm the interaction between CKB and cytoskeletal proteins such as ARP2. Second, the hypothesis that *Ckb* is a transcriptional target of YAP-TEADs needs validation *via* ChIP and luciferase assays. We acknowledge that direct validation of our specific phenotypic observations in a cell culture setting would further strengthen the generalizability of our conclusions. Notably, the convergence of our findings with an independent study in human pancreatic cancer (51) suggests that the coupling between creatine metabolism and YAP signaling may represent a fundamental cellular mechanism—a concept warranting further exploration in other systems.

**Figure 7:**
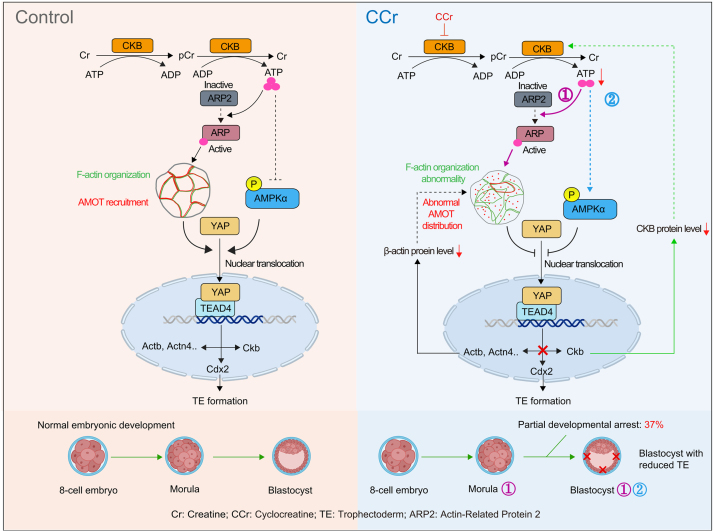
**Graphical Abstract.** Created in BioRender.

In conclusion, this study systematically elucidates the central role and underlying mechanisms of creatine metabolism in regulating TE formation and blastocyst development in mouse early embryos (Fig. 7). Our study functionally demonstrates that from the 8-cell stage onward, the embryo utilizes the creatine metabolism system to maintain local ATP homeostasis, ensuring proper assembly of F-actin, which subsequently relieves the inhibition on the Hippo pathway effector YAP and promotes its nuclear translocation. The activated YAP-TEADs complex then drives a transcriptional program encompassing cytoskeletal genes, creatine metabolic enzymes, and TE fate determinants, thereby precisely orchestrating TE formation. The discovery of this "energy-cytoskeleton-YAP" axis in early embryonic development integrates a fundamental metabolite, cytoskeletal dynamics, and a core developmental signaling pathway. This work not only fills a gap in the field of metabolic regulation during early embryogenesis but also provides a novel theoretical framework for understanding how metabolism-environment interactions influence reproductive health.

## Experimental procedures

### Animals and ethics statement

All experimental procedures were strictly conducted in accordance with the regulations of the animal ethics and welfare committee (AEWC) of Sichuan Agricultural University, China (Approval Code: AEWC2016, dated January 6, 2016).

### Oocyte collection

Kunming White female and male mice (10 weeks old) were purchased from Chengdu Dashuo Experimental Animal Co., Ltd. The mice were maintained under a 14:10 light/dark cycle (lights on from 06:00 to 20:00), with the ambient temperature controlled between 18 and 25 °C and humidity maintained at 50 to 70%. After a 2-week acclimation period, the mice were used for experiments. For superovulation, each female mouse was intraperitoneally injected with 5 IU of pregnant mare serum gonadotropin (PMSG, Ningbo Second Hormone Factory). After 48 h, they were injected with 5 IU of human chorionic gonadotropin (hCG, Ningbo Second Hormone Factory). The mice were euthanized by cervical dislocation at 14 to 16 h post-hCG injection. Their oviducts were collected, and cumulus-oocyte complexes (COCs) were retrieved for subsequent use.

### *In vitro* fertilization (IVF) and *in vitro* embryo culture (IVC)

IVF: Male mice were euthanized by cervical dislocation, and the epididymis was collected. Sperm were gently extruded and placed in a 200 μl droplet of HTF medium covered with mineral oil. The sperm suspension was then incubated at 37 °C in a 5% CO_2_ incubator for 1 h to achieve capacitation. Prior to the completion of capacitation, female mice that had been injected with hCG 14 to 16 h earlier were euthanized, and their oviducts were collected. COCs were released by gentle tearing of the oviducts with a syringe needle and transferred into pre-prepared fertilization droplets. Approximately 1 μl of motile sperm was added to each fertilization droplet. Gametes were co-incubated for 4 to 6 h at 37 °C under 5% CO_2_.

IVC: After fertilization, zygotes were washed three times in pre-equilibrated IVC droplets and then transferred to clean IVC droplets for further culture. Cleavage, 4-cell, 8-cell, morula, and blastocyst rates were calculated at 24, 48, 55, 72, and 96 hpi (hours post-insemination), respectively.

### Treatment with Cr, CCr, dorsomorphin, GA-017, 2-deoxy-D-glucose, and CK666

Cr (1 mmol/L, Sigma, C0780); CCr (0.25, 0.5, and 1 mmol/L, Med Chem Express, HY-W017540), as a creatine metabolism inhibitor (67, 68); Dorsomorphin (1, 5, and 10 μmol/L, Med Chem Express, HY-13418A), an AMPK inhibitor that reduces cellular p-AMPK levels (69, 70); GA-017 (1, 5, and 10 μmol/L, Med Chem Express, HY-147082), a YAP agonist that promotes YAP nuclear localization (71); 2-deoxy-D-glucose (5 μmol/L, Med Chem Express, HY-13966), a glycolysis inhibitor that reduces intracellular ATP levels (72, 73), and CK666 (50, 100, and 200 μmol/L, Med Chem Express, HY-16926), an inhibitor of the Arp2/3 complex that stabilizes its inactive state, thereby preventing the movement of the Arp2 and Arp3 subunits into the activated filamentous conformation.

*In vitro* treatment with CCr or Cr: the addition times for treatments from zygote to blastocyst, from 8-cell to blastocyst, and from morula to blastocyst were 6 to 96, 55 to 96, and 72 to 96 hpi, respectively; treatment from zygote to 8-cell stage was performed from 6 to 55 hpi, followed by continued culture to the blastocyst stage.

*In vitro* treatment with Dorsomorphin, GA-017, 2-deoxy-D-glucose, and CK666: Dorsomorphin and GA-017 were added to IVC medium at 55 hpi simultaneously with CCr. 2-deoxy-D-glucose and CK666 were added to the IVC medium at 55 hpi. Embryos were then cultured until the blastocyst stage.

*In vivo* CCr treatment: following mating of female and male mice, females were randomly divided into two groups (n = 4 per group). The treatment group received daily intraperitoneal injections of CCr (285 mg/kg) (74), while the control group received an equal volume of normal saline. Injections were administered for three consecutive days, and blastocysts were collected on the fourth day.

### Immunofluorescence staining

Embryos were first fixed in 4% paraformaldehyde solution for 20 min, then transferred to a washing buffer (PBS solution containing 0.1% Triton X-100 and 0.01% Tween 20) for 10 min. Following this, they were permeabilized in a permeabilization solution (PBS solution containing 1% Triton X-100) at room temperature for 20 min. After permeabilization, the embryos were transferred to a blocking solution (PBS solution containing 1% BSA) and blocked at room temperature for 30 min. They were then incubated with the primary antibody overnight at 4 °C. After primary antibody incubation, the embryos were washed three times in washing buffer, followed by incubation with a fluorescently labeled secondary antibody at room temperature for 1 h. After secondary antibody incubation, the embryos were washed three times in washing buffer, then stained with DAPI-containing anti-fade mounting medium (Vector Laboratories Inc) for 1 min, washed in washing buffer for 10 min, and finally mounted on glass slides with coverslips. Images were acquired using a laser scanning confocal microscope (FEV4000-IX83, Olympus). Fluorescence intensity for each embryo was quantified using ImageJ software (Version 1.48, National Institutes of Health).

The detailed information for antibodies used in immunofluorescence staining is as follows: primary antibodies included SOX2 (Cell Signaling Technology, 23064S, 1:200), CDX2 (Biogenex, AM392-5M, ready-to-use), YAP (Cell Signaling Technology, 14074T, 1:200), AMOT (Cell Signaling Technology, 30020T, 1:200), N-WASP (Proteintech, 14306-1-AP, 1:200) and ARP2 (Proteintech, 10922-1-AP, 1:200); directly conjugated antibodies were F-actin (Proteintech, PF00003/PF00001, 1:1000) and α-tubulin (Sigma, F2168, 1:500); secondary antibodies comprised CoraLite488-conjugated Goat Anti-rabbit IgG (Proteintech, SA00013-2, 1:400), CoraLite594-conjugated Goat Anti-rabbit IgG (Proteintech, SA00013-4, 1:400), CoraLite594-conjugated Goat Anti-mouse IgG (Proteintech, SA00013-3, 1:400), and Alexa Fluor 647-conjugated Donkey anti-rabbit IgG (Thermo Fisher Scientific, A-31573, 1:1000). Antibody specificity was validated by the manufacturer using knockout cell lines or molecular weight confirmation.

### Protein synthesis assay

Protein synthesis levels in mouse blastocysts were assessed using the Click-iT Protein Synthesis Assay Kit (Thermo Fisher Scientific, C10428). The blastocysts were transferred into methionine-free medium containing 50 μM L-homopropargylglycine (HPG) and incubated at 37 °C for 1 h. Subsequently, they were fixed with 4% paraformaldehyde at room temperature for 30 min, washed in washing buffer (PBS containing 0.1% Triton X-100 and 0.01% Tween 20) for 10 min, and permeabilized in permeabilization solution (PBS containing 1% Triton X-100) at room temperature for 20 min. Next, the embryos were incubated in the reaction cocktail solution (Thermo Fisher Scientific, C10428) provided in the kit at room temperature for 30 min in the dark, and then washed in washing buffer for 10 min. The embryos were then stained with DAPI-containing anti-fade mounting medium (Vector Laboratories Inc) for 1 min, washed in washing buffer for 10 min, and finally mounted on glass slides with coverslips. Images were acquired using a laser scanning confocal microscope (FEV4000-IX83, Olympus). Fluorescence intensity for each embryo was quantified using ImageJ software (Version 1.48, National Institutes of Health).

### ATP level and distribution detection

Kit: ATP levels were measured according to the manufacturer’s instructions (A095-2, Nanjing Jiancheng Bioengineering Institute). First, the collected morulae and blastocysts were washed three times in M2 medium. Subsequently, 10 embryos were transferred into 20 μl of lysis buffer and kept on ice for further processing. Next, the enzyme working solution was prepared following the protocol instructions, and the assay was performed according to the operational requirements. Subsequently, fluorescence values were detected using a chemiluminescence detection instrument (Varioskan Lux, Thermo). Finally, a standard curve was generated based on nine ATP concentration standards, and the ATP concentration in the samples was calculated according to the standard curve. The concentration range was 0 to 2 μmol/L.

Fluorescent probe staining: embryos were fixed in 4% paraformaldehyde at room temperature for 1 h, then transferred to PBS solution containing 1% PVA and washed for 10 min. Subsequently, they were incubated in BODIPY FL ATP dye (Thermo Fisher Scientific, A12410, 0.5 μM) at room temperature for 1 h in the dark. After incubation, the embryos were washed three times. Finally, they were mounted on glass slides, coverslipped, and imaged using a laser scanning confocal microscope (FEV4000-IX83, Olympus).

### Quantitative reverse transcription PCR (qRT-PCR)

Blastocysts (≥10 per group) were first collected, and total cDNA was obtained using the TransScript-Uni Cell to cDNA Synthesis SuperMix for Q-PCR kit (TransGen Biotech). Subsequently, quantitative analysis of cDNA was performed using the TransStart Tip Green qPCR SuperMix kit (TransGen Biotech) on a CFX Connect Real-Time Detection System (Bio-Rad). The relative mRNA expression levels were calculated using the 2^−ΔΔct^ method, with 18S rRNA serving as the internal reference gene for normalization. The primer sequences used are listed in Table S1.

### Western blot

The samples were collected by transferring 100 morulae or blastocysts per group into RIPA lysis buffer (AR 0102, BOSTER) containing protease inhibitor (AR 1182-1, BOSTER) and phosphatase inhibitor (AR 1183, BOSTER), followed by lysis on ice for 10 min. Subsequently, 5 × SDS-PAGE loading buffer was added, and the mixture was boiled at 95 °C for 10 min. The protein samples were then separated by SDS-PAGE and transferred onto a PVDF membrane (FFP32, Beyotime). The membranes were blocked in blocking buffer at room temperature for 2 h. The membranes were then incubated with primary antibodies overnight at 4 °C: HK2 (Cell Signaling Technology, 2867T, 1:1000); GAPDH (Proteintech, 10494-1-AP, 1:5000); ENO1 (Cell Signaling Technology, 3810T1, 1:1000); PKM1/2 (Cell Signaling Technology, 3190T, 1:1000); active-YAP (Cell Signaling Technology, 29495T, 1:1000); AMPK (Cell Signaling Technology, 5831T, 1:1000); p-AMPK (Cell Signaling Technology, 2535T, 1:1000); β-actin (Proteintech, PTG-66009-1, 1:5000); CKB (Proteintech, 15137-1-AP, 1:5000); β-tubulin (Proteintech, PTG-80713-1, 1:5000). Antibody specificity was validated by the manufacturer using knockout cell lines or molecular weight confirmation. The next day, after washing, the membranes were incubated with HRP-conjugated secondary antibodies for 1 h at room temperature: goat anti-rabbit (Proteintech, SA00001-2, 1:5000) and goat anti-mouse (Proteintech, SA00001–1, 1:5000). Following another three washes with TBST, the membranes were processed using ECL (Yeasen, 36208ES60) and detected with a protein immunoblot imaging system (Tanon Science & Technology Co., Ltd). Grayscale values were quantified using ImageJ software. Normalization was performed using β-tubulin as an internal reference.

### T&T-seq protocol and data analysis for mouse embryos

T&T-seq Procedure: morulae and blastocysts from each group after *in vitro* fertilization were collected and incubated in Acidic Tyrode’s Solution (T1788, Sigma-Aldrich) for 30 s to remove the zona pellucida. The zona-free embryos were then washed twice with PBS and transferred into 10 μl of cell lysis buffer (N712, Vazyme). After lysis on ice for 10 min, the lysate was divided into two aliquots for transcriptome and translatome sequencing, respectively. For translatome analysis, ribosome-associated mRNA was isolated using RiboLace magnetic beads (RL001, Immagina), purified, and subjected to reverse transcription and cDNA amplification with the Single-Cell Full-Length mRNA Amplification Kit (N712, Vazyme). Libraries were constructed using the TruePrep DNA Library Prep Kit V2 (TD502/503, Vazyme) and sequenced on an Illumina NovaSeq 6000 platform.

Data Analysis: differentially expressed genes (DEGs) were identified using DESeq2 software, with the screening criteria set as *p* < 0.05 and fold change > 1.5. Kyoto Encyclopedia of Genes and Genomes (KEGG) enrichment analysis of DEGs with coordinated upregulation or downregulation at both transcriptional and translational levels was performed using the Metascape software.

### Statistical analysis

Statistical analyses were performed using unpaired two-tailed *t* test and one-way ANOVA followed by Fisher’s LSD *post hoc* test with SPSS software. Data are presented as mean ± SD of three independent experiments. The number of biological replicates was equal to or greater than three. Prior to analysis, all data underwent normality distribution and homogeneity of variance tests. Percentage data were arcsine-transformed before conducting ANOVA. For all analyses, *p* < 0.05 was considered statistically significant. Different letters (a, b, c, d) indicate statistically significant differences between groups, while the same letter indicates no significant difference between groups.

## Data availability

T&T-seq data of mice generated in this study have been deposited at the Genome Sequence Archive and are publicly available at the date of publication. Accession number is CRA036761. Any additional information required to reanalyze the data reported in this paper is available from the lead contact upon request.

## Supporting information

This article contains supporting information.

## Conflict of interest

The authors declare that they have no conflicts of interest with the contents of this article.

## Figures and Tables

**Figure S6:**