# Supplementary material for: Creatine metabolism regulates trophectoderm formation in early mouse embryos via an energy-cytoskeleton-YAP axis
Source: J Biol Chem. 2026 May 18;302(7):113147. doi: 10.1016/j.jbc.2026.113147 (PMC13264175; doi:10.1016/j.jbc.2026.113147)
Supplement: Supporting Information [file mmc1.doc]

*Supporting Information*

Creatine metabolism regulates trophectoderm formation in early mouse embryos via an energy-cytoskeleton-YAP axis

Xiangyi Chen^1,#^, Bo Pan^1,#^, Jianpeng Qin^1^, Yaozong Wei^1^, Kunlin Du^1^, Ao Ning^1^, Tao Sun^1^, Can Wang^1^, Shuqi Zou^1^, Xiaohui Su^1^, Mengying Wang^1^, Yinan Guo^1^, Xiaoyue Xiong^2^, Shiyuan Pei^2^, Shengqin Zang^1^, Chang Zhang^1^, Jiangfeng Ye^1^, Guozhi Yu^2^, Qiuxia Liang^2,*^, Guangbin Zhou^1,*^

^1^ State Key Laboratory of Swine and Poultry Breeding Industry, Key Laboratory of Livestock and Poultry Multiomics, Ministry of Agriculture and Rural Affairs, Farm Animal Genetic Resources Exploration and Innovation Key Laboratory of Sichuan Province, College of Animal Science and Technology, Sichuan Agricultural University, Chengdu 611130, China.

^2^ College of Life Science, Sichuan Agricultural University, Ya'an 625014, China.

# Contribute equally

* Correspondence: Qiuxia Liang ([liangqx@sicau.edu.cn](mailto:liangqx@sicau.edu.cn)), Guangbin Zhou (zguangbin@sicau.edu.cn)

**This PDF file includes:**

Supplementary Text

Figs. S1 to S6

Tables S1 to S3.


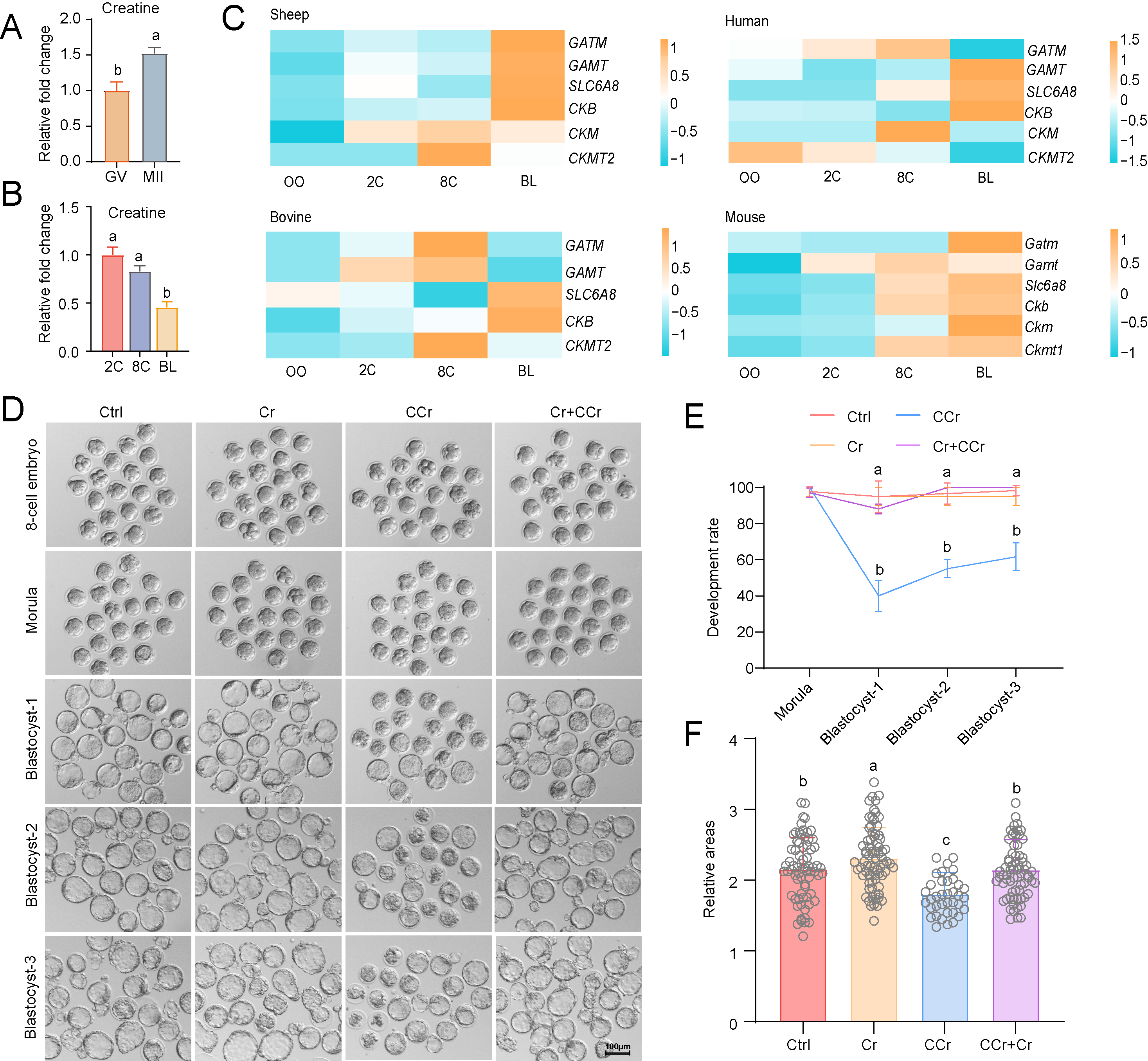
**F****igure S1.** **Inhibition of creatine metabolism leads to delayed development or arrest in a subset of embryos following the morula stage.** (A–B*)* Dynamic changes in creatine levels during sheep oocyte maturation and early embryo development, respectively. (C) Expression dynamics of creatine metabolism-related genes (*Slc6a8* and *Ckb*) during early embryo development in sheep, human, cattle, and mouse. (D) Representative images of 8-cell embryos, morulae, and blastocysts in each group. Scale bar: 100 µm. (E) Embryo development rates in each group. (F) Blastocyst area on day 4 of *in vitro* culture for each group. Control (n = 59), Cr (n = 60), CCr (n = 60), and rescue (n = 59). Blastocyst-1, -2, and -3 correspond to blastocysts cultured for 4, 4.5, and 5 days, respectively. Error bars represent mean ± SD. Different letters (a, b, c, d) indicate statistically significant differences between groups (*p* < 0.05), exact *p*-values are provided in Table S3. Data were analyzed by one-way ANOVA followed by Tukey’s multiple comparison test. *N* = 3 independent experiments for (E, F).


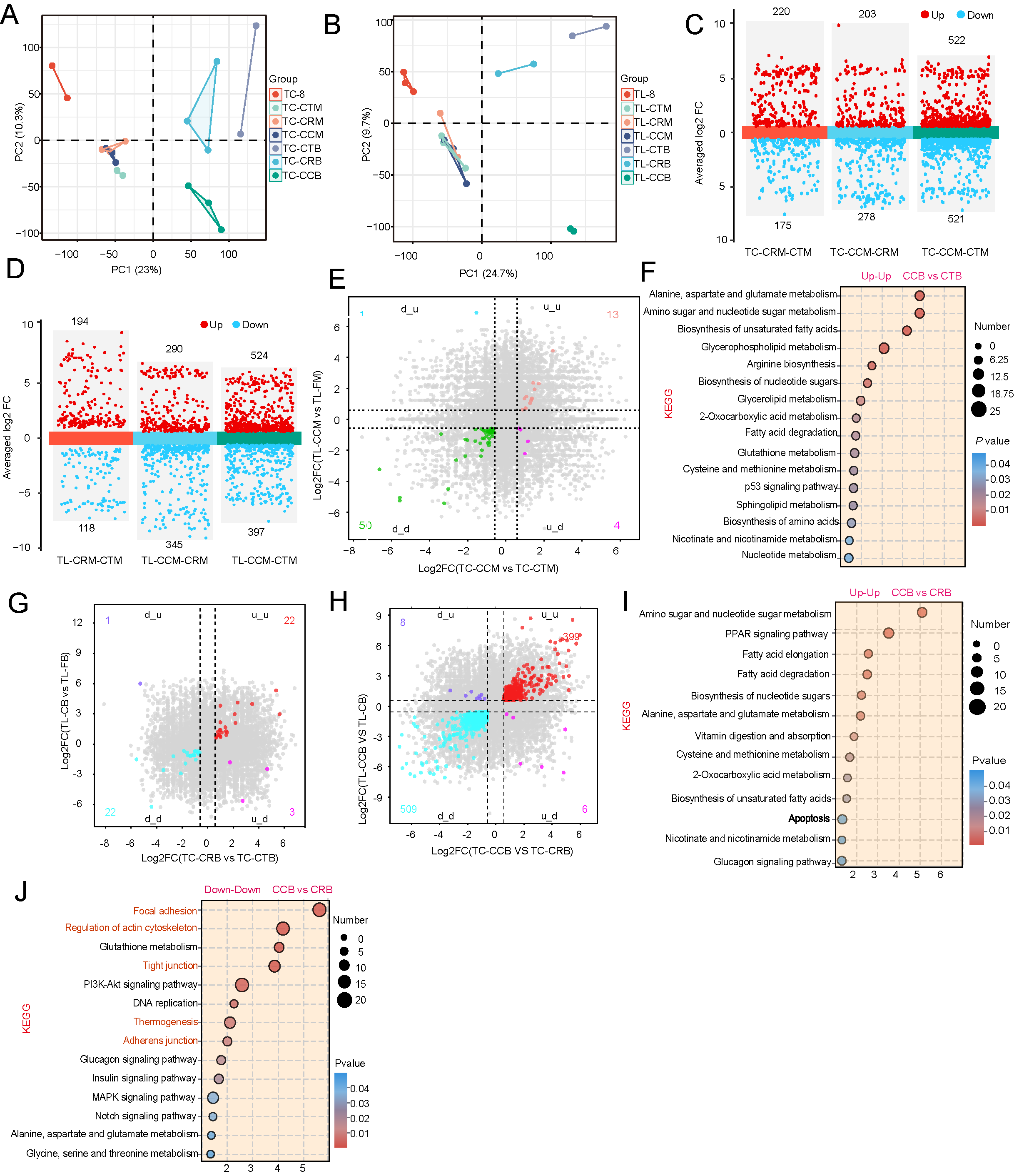
**Figure S2. Differential genes and pathway enrichment analysis among different treatment groups.** (A‒B) Principal component analysis (PCA) plots for transcriptome and translatome data, respectively. (C‒D) Volcano plots of differentially expressed genes in the transcriptome and translatome at the morula stage. (E) Nine-quadrant diagram displaying genes coordinately upregulated or downregulated in both transcriptome and translatome in the CCr group compared to the control group. (F) KEGG pathway enrichment analysis of the coordinately upregulated genes from Fig. 3D. (G‒H) Nine-quadrant diagrams showing genes coordinately upregulated or downregulated in both transcriptome and translatome in the Cr group compared to the control group (G), and in the CCr group compared to the Cr group (H), respectively. (I‒J) KEGG pathway enrichment analysis based on the coordinately upregulated (I) and downregulated (J) genes from (H). CTB, control group blastocyst; CRB, creatine group blastocyst; CCB, cyclocreatine group blastocyst. CTM, control group morula; CRM, creatine group morula; CCM, cyclocreatine group morula.


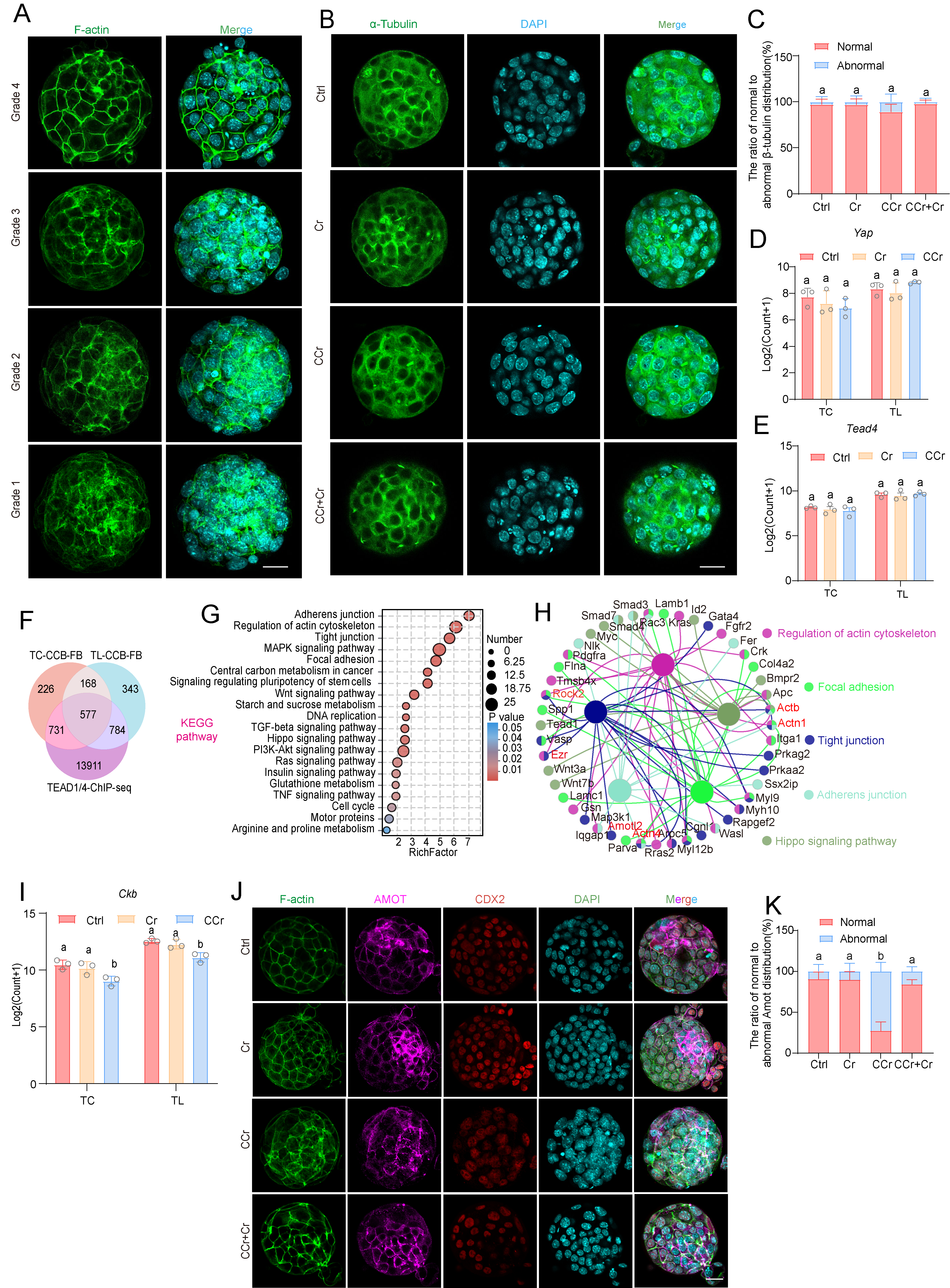
**Figure S3. The effects of inhibiting creatine metabolism on microtubules, TEADs function, and AMOT in blastocysts.** (A) Criteria for normal vs. abnormal F-actin in morulae. Scale bar: 50 μm. (B) Immunofluorescence staining images of α-tubulin and DAPI in blastocysts from each group. Scale bar: 50 μm. (C*)* Proportion of blastocysts with normal vs. abnormal α-tubulin distribution in each group. Control (n = 42), Cr (n = 32), CCr (n = 34), rescue (n = 43). (D–E) Transcriptomic and translatomic counts of the *Yap* and *Tead4* genes in blastocysts from each group. (F) Venn diagram intersecting downregulated differentially expressed genes from both transcriptome and translatome in CCr-treated blastocysts with public TEAD1/4 ChIP-seq data (https://doi.org/10.1093/nar/gkac624). (G) KEGG pathway enrichment analysis of the 577 overlapping genes identified in (F). (H) Gene network diagram of the enriched pathways from (G). (I) Transcriptomic and translatomic counts of the *Ckb* gene in blastocysts from each group. (J) Immunofluorescence staining images of F-actin, AMOT, CDX2, and DAPI in blastocysts from each group. Scale bar: 50 μm. (K) Proportion of blastocysts with normal vs. abnormal AMOT protein distribution in each group. Control (n = 58), Cr (n = 58), CCr (n = 54), rescue (n = 60). Error bars represent mean ± SD. Different letters (a, b, c, d) indicate statistically significant differences between groups (*p* < 0.05), exact *p*-values are provided in Table S3. Data were analyzed by one-way ANOVA followed by Tukey’s multiple comparison test. *N* = 3 independent experiments for (C, K).


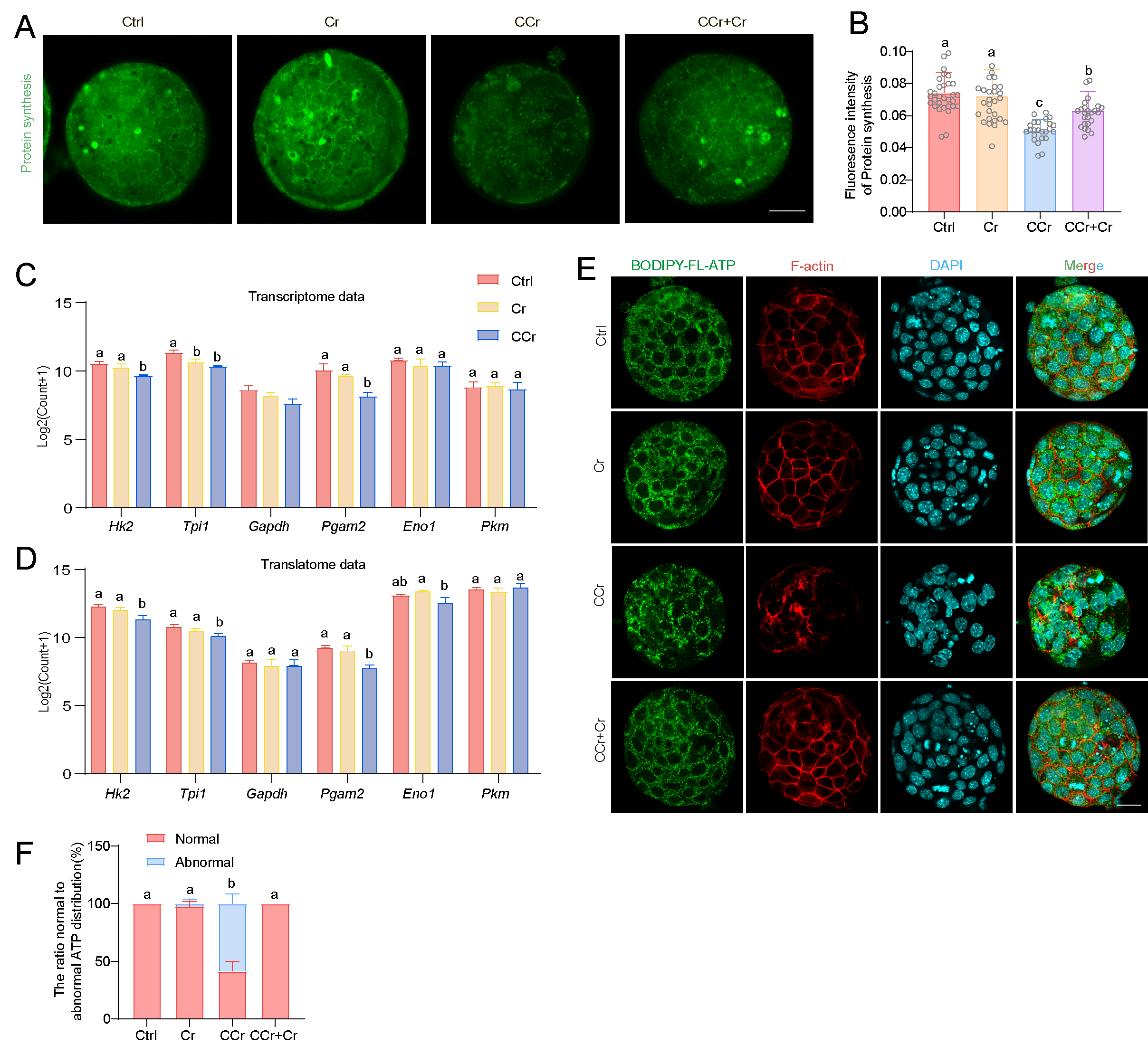
**Figure S4. The effects of inhibiting creatine metabolism on nascent protein synthesis, glycolytic genes, and ATP distribution.** (A) Immunofluorescence staining images of the nascent protein synthesis marker (HPG) in blastocysts from each group. Scale bar: 50 μm. (B) Mean fluorescence intensity of HPG in blastocysts from each group. Control (n = 32), Cr (n = 30), CCr (n = 24), and rescue (n = 25). (C–D) Transcriptomic and (C) and translatomic (D) counts of the *Hk2*, *Tpi1*, *Gapdh*, *Pgam2*, *Eno1*, and *Pkm* genes in blastocysts from each group. (E) Immunofluorescence staining images of ATP, F‑actin, and DAPI in blastocysts from each group. Scale bar: 50 μm. (F) Proportion of blastocysts showing normal vs. abnormal ATP distribution in each group. Control (n = 38), Cr (n = 36), CCr (n = 36), and rescue (n = 39). Error bars represent mean ± SD. Different letters (a, b, c, d) indicate statistically significant differences between groups (*p* < 0.05), exact *p*-values are provided in Table S3. Data were analyzed by one-way ANOVA followed by Tukey’s multiple comparison test. *N* = 3 independent experiments for (B, F).


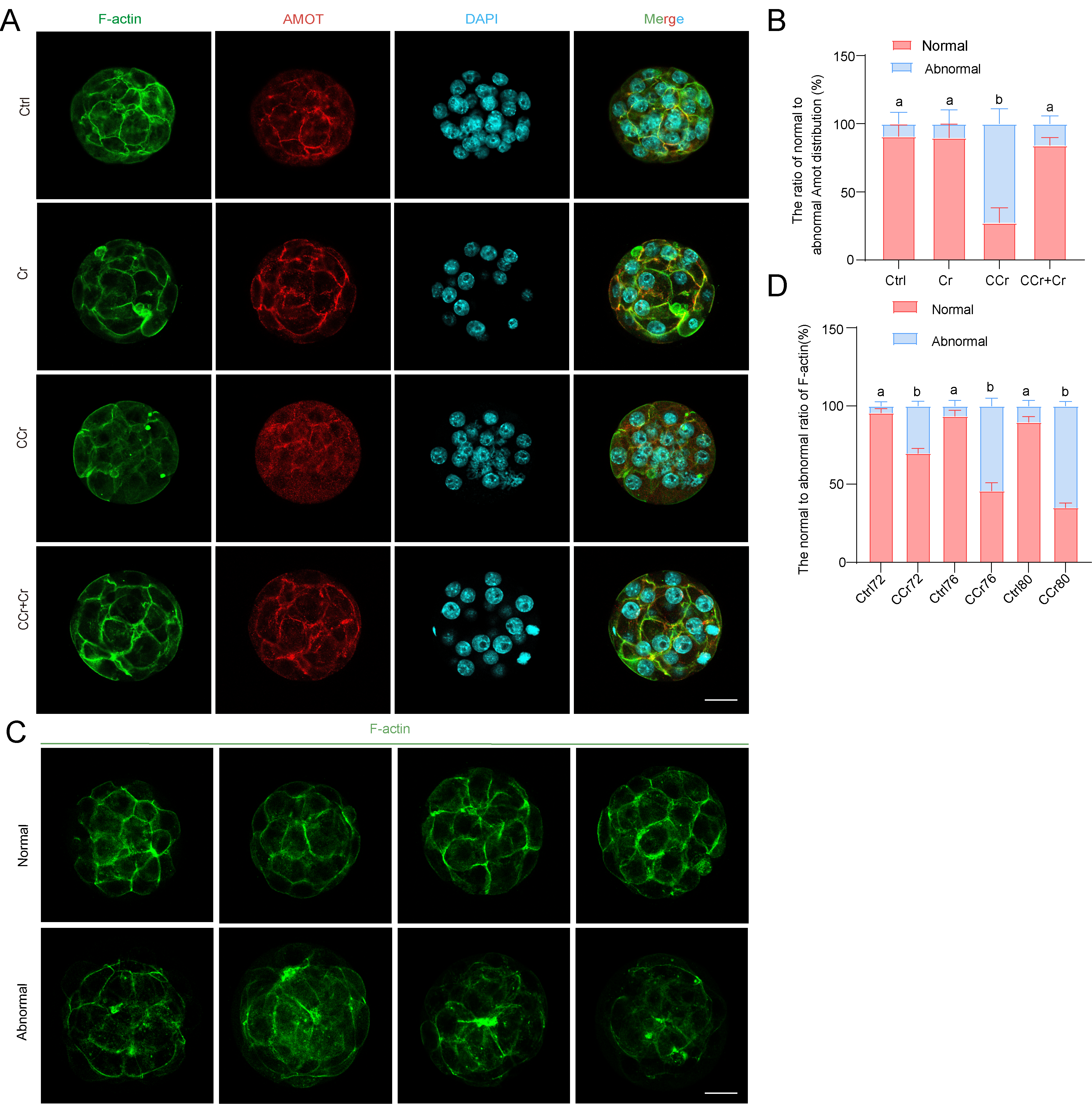
**Figure S5. The effects of inhibiting creatine metabolism on AMOT distribution and F-actin dynamics at different time points.** (A) Immunofluorescence staining images of F‑actin, AMOT, and DAPI in morulae from each group. Scale bar: 50 μm. (B) Proportion of morulae showing normal vs. abnormal AMOT protein distribution in each group. Control (n = 30), Cr (n = 28), CCr (n = 29), and rescue (n = 25). (C) Evaluation criteria for classifying normal vs. abnormal F‑actin in morulae. (D) Proportion of morulae with normal vs. abnormal F‑actin in the control and CCr groups at 72, 76, and 80 hpi. Error bars represent mean ± SD. Different letters (a, b, c, d) indicate statistically significant differences between groups (*p* < 0.05), exact *p*-values are provided in Table S3. Data were analyzed by one-way ANOVA followed by Tukey’s multiple comparison test for (B), and Student’s t test for (D). *N* = 3 independent experiments for (B, D). Hpi, hours post-insemination.

**Figure S6.** **Protein interaction network of CKB and creatine metabolism inhibition on N-WASP distribution in morulae.** (A–F) Network diagrams of
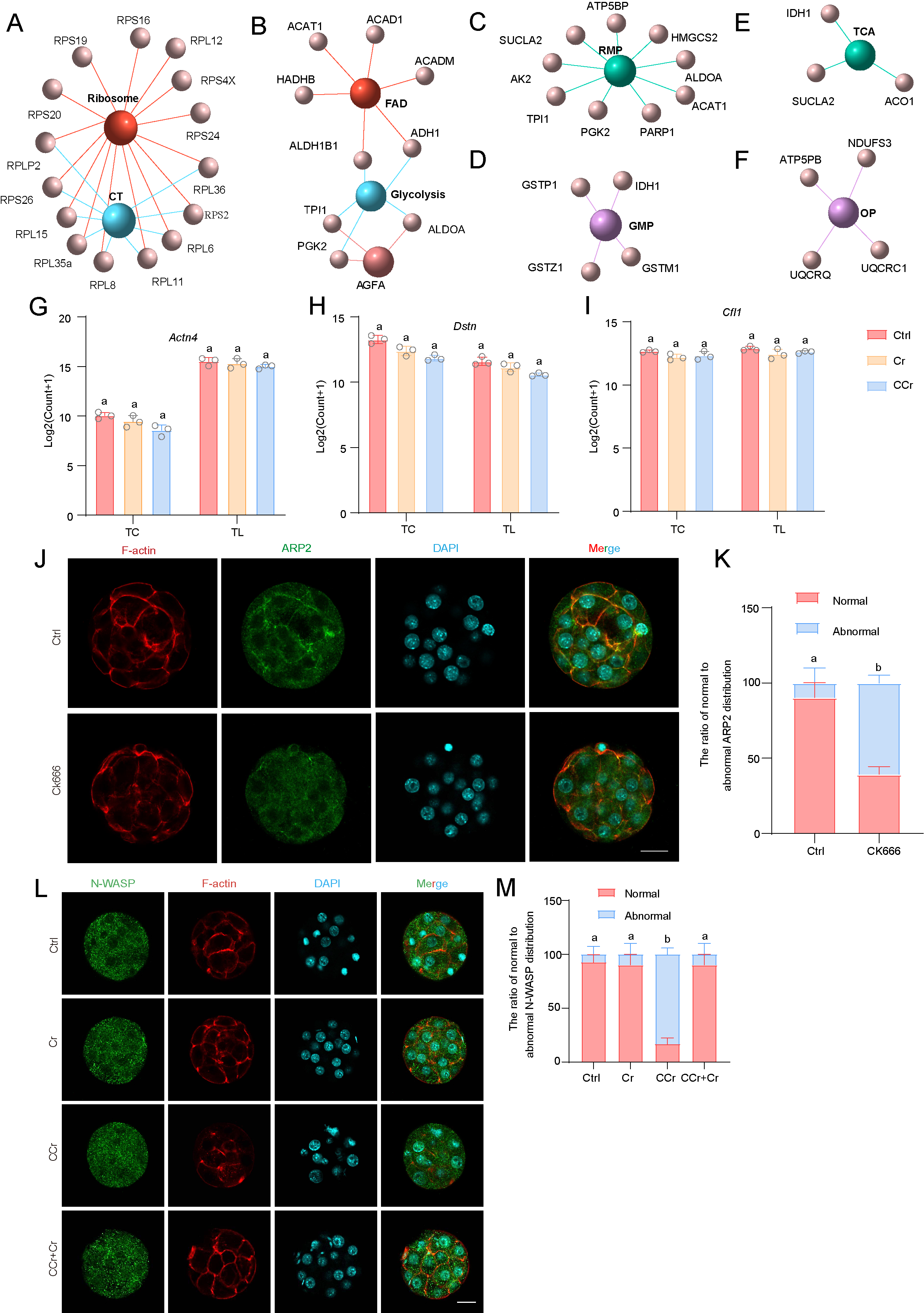
 proteins potentially interacting with CKB, enriched in pathways related to ribosome and cytoplasmic translation (A), fatty acid degradation and glycolysis (B), ribonucleotide biosynthesis (C), glutathione metabolism (D), the tricarboxylic acid cycle (E), and oxidative phosphorylation (F), respectively. (G-I) Translatomic counts of *Actn4*, *Dstn*, and *Cfl1* genes in morulae from each group, respectively. (J) Immunofluorescence staining images of ARP2, F-actin, and DAPI in morulae from the control group and the 200 μM CK666 group. Scale bar: 50 μm. (K) Proportion of normal versus abnormal ARP2 distribution in morulae in each group. Control (n = 25), CCr (n = 37), CK666 (n = 38). (L) Immunofluorescence staining images of N-WASP, F‑actin, and DAPI in morulae from each group. Scale bar: 50 μm. (M) Proportion of morulae showing normal vs. abnormal N-WASP protein distribution in each group. Control (n = 40), Cr (n = 30), CCr (n = 30), and rescue (n = 30). Error bars represent mean ± SD. Different letters (a, b, c, d) indicate statistically significant differences between groups (*p* < 0.05), exact *p*-values are provided in Table S3. Data were analyzed by one-way ANOVA followed by Tukey’s multiple comparison test for (M), and Student’s t test for (K). *N* = 3 independent experiments for (K, M).

**Table S1. Primers for RT-qPCR used in this study.**

| **Genes** | **Primer sequences** | **GenBank number** | **Tm(◦C)** |
| --- | --- | --- | --- |
| *Sox2* | GCGGAGTGGAAACTTTTGTCC | NM_011443.4 | 60 |
|  | CGGGAAGCGTGTACTTATCCTT |  |  |
| *Cdx2* | CAAGGACGTGAGCATGTATCC | NM_007673.3 | 60 |
|  | GTAACCACCGTAGTCCGGGTA |  |  |
| *Pcna* | TTTGAGGCACGCCTGATCC | NM_011045.2 | 60 |
|  | GGAGACGTGAGACGAGTCCAT |  |  |
| *Elf5* | ATTCGCTCGCAAGGTTACTCC | NM_010125.3 | 60 |
|  | GGATGCCACAGTTCTCTTCAG |  |  |
| *Gata3* | CTCGGCCATTCGTACATGGAA | NM_008091.4 | 60 |
|  | GGATACCTCTGCACCGTAGC |  |  |
| *Ckb* | AGTTCCCTGATCTGAGCAGC | NM_021273.4 | 60 |
|  | GAATGGCGTCGTCCAAAGTAA |  |  |
| *Hk2* | TGCCACCAGACTAAACTAGACG | NM_013820.4 | 60 |
|  | CCCGTGCCCACAATGAGAC |  |  |
| *Gapdh* | CATGGCCTTCCGTGTTCCTA | NM_008084.3 | 60 |
|  | GCCTGCTTACCACCTTCTT |  |  |
| *Pgam2* | GTCCTTATTGCAGCCCATGG | NM_018870.3 | 60 |
|  | ACCCACTCTCACTTTGCCTT |  |  |
| *Tpi1* | CCAGGAAGTTCTTCGTTGGGG | NM_009415.3 | 60 |
|  | CAAAGTCGATGTAAGCGGTGG |  |  |
| *Eno1* | TGCGTCCACTGGCATCTAC | NM_023119.3 | 60 |
|  | CAGAGCAGGCGCAATAGTTTTA |  |  |
| *Pkm* | TGCCGCCTGGACATTGACTC | NM_001253883.2 | 60 |
|  | ATTCAGCCGAGCCACATTCATTC |  |  |
| *18S rRNA* | CGGCGACGACCCATTCGAAC | - | 60 |
|  | GAATCGAACCCTGATTCCCCGTC |  |  |

**Table S2. Summary of pairwise comparison *p*-values for main figures.**

| **Figure panel** | **Comparison groups** | ***p*-value** |
| --- | --- | --- |
| Fig. 1C | control vs. 0.25 mM CCr | *p* < 0.001 |
| Fig. 1C | control vs. 0.5 mM CCr | *p* < 0.001 |
| Fig. 1C | control vs. 1 mM CCr | *p* < 0.001 |
| Fig. 1E | control vs. CCr | *p* = 0.008 |
| Fig. 1G | control vs. CCr | *p* = 0.117 |
| Fig. 1H | control vs. CCr | *p* = 0.027 |
| Fig. 1J | control vs. CCr | *p* = 0.39 |
| Fig. 1M | control vs. CCr | *p* = 0.005 |
| Fig. 2C | control vs. Cr | *p* = 0.299 |
| Fig. 2C | control vs. CCr | *p* = 0.014 |
| Fig. 2C | CCr vs. CCr. + Cr | *p* = 0.007 |
| Fig. 2C | control vs. CCr + Cr | *p* = 0.649 |
| Fig. 2E | control vs. Cr | *p* = 0.995 |
| Fig. 2E | control vs. CCr | *p* < 0.001 |
| Fig. 2E | CCr vs. CCr + Cr | *p* = 0.001 |
| Fig. 2E | control vs. CCr + Cr | *p* = 0.425 |
| Fig. 2F | control vs. Cr | *p* = 0.011 |
| Fig. 2F | control vs. CCr | *p* = 0.084 |
| Fig. 2F | CCr vs. CCr + Cr | *p* = 0.002 |
| Fig. 2F | control vs. CCr + Cr | *p* = 0.197 |
| Fig. 2G | control vs. Cr | *p* = 0.088 |
| Fig. 2G | control vs. CCr | *p* < 0.001 |
| Fig. 2G | CCr vs. CCr + Cr | *p* < 0.001 |
| Fig. 2G | control vs. CCr + Cr | *p* = 0.053 |
| Fig. 2H (*Sox2*) | control vs. Cr | *p* = 0.172 |
| Fig. 2H (*Sox2*) | control vs. CCr | *p* = 0.155 |
| Fig. 2H (*Sox2*) | CCr vs. CCr + Cr | *p* = 0.009 |
| Fig. 2H (*Sox2*) | control vs. CCr + Cr | *p* = 0.101 |
| Fig. 2H (*cdx2*) | control vs Cr | *p* = 0.006 |
| Fig. 2H (*cdx2*) | control vs. CCr | *p* < 0.001 |
| Fig. 2H (*cdx2*) | CCr vs. CCr + Cr | *p* < 0.001 |
| Fig. 2H (*cdx2*) | control vs. CCr + Cr | *p* < 0.001 |
| Fig. 2H (*Pcna*) | control vs. Cr | *p* = 0.023 |
| Fig. 2H (*Pcna*) | control vs. CCr | *p* = 0.038 |
| Fig. 2H (*Pcna*) | CCr vs. CCr + Cr | *p* < 0.001 |
| Fig. 2H (*Pcna*) | control vs. CCr + Cr | *p* = 0.097 |
| Fig. 2I (*Elf5*) | control vs. Cr | *p* = 0.588 |
| Fig. 2I (*Elf5*) | control vs. CCr | *p* = 0.002 |
| Fig. 2I (*Elf5*) | CCr vs. CCr + Cr | *p* < 0.001 |
| Fig. 2I (*Elf5*) | control vs. CCr + Cr | *p* = 0.675 |
| Fig. 2I (*Gata3*) | control vs. Cr | *p* = 0.644 |
| Fig. 2I (*Gata3*) | control vs. CCr | *p* = 0.014 |
| Fig. 2I (*Gata3*) | CCr vs. CCr + Cr | *p* = 0.03 |
| Fig. 2I (*Gata3*) | control vs. CCr + Cr | *p* = *0.635* |
| Fig. 2K | control vs. CCr | *p* < 0.001 |
| Fig. 2L | control. vs. CCr | *p* < 0.001 |
| Fig. 2M | control vs. CCr | *p* < 0.001 |
| Fig. 3G | control vs. Cr | *p* = 0.818 |
| Fig. 3G | control vs. CCr | *p* < 0.001 |
| Fig. 3G | CCr vs. CCr + Cr | *p* = 0.008 |
| Fig. 3G | control vs. CCr + Cr | *p* = 0.238 |
| Fig. 3H | control vs. Cr | *p* = 0.677 |
| Fig. 3H | control vs. CCr | *p* < 0.001 |
| Fig. 3H | CCr vs. CCr + Cr | *p* < 0.001 |
| Fig. 3H | control vs. CCr + Cr | *p* = 0.696 |
| Fig. 3I | control vs. Cr | *p* = 0.872 |
| Fig. 3I | control vs. CCr | *p* = 0.009 |
| Fig. 3I | CCr vs. CCr + Cr | *p* < 0.001 |
| Fig. 3I | control vs. CCr + Cr | *p* = 0.131 |
| Fig. 3K | control vs. Cr | *p* = 0.717 |
| Fig. 3K | control vs. CCr | *p* < 0.001 |
| Fig. 3K | CCr vs. CCr + Cr | *p* < 0.001 |
| Fig. 3K | control vs. CCr + Cr | *p* = 0.009 |
| Fig. 3L | control vs. Cr | *p* = 0.453 |
| Fig. 3L | control vs. CCr | *p* = 0.005 |
| Fig. 3L | CCr vs. CCr + Cr | *p* = 0.002 |
| Fig. 3L | control vs. CCr + Cr | *p* = 0.612 |
| Fig. 3M | control vs. Cr | *p* = 0.533 |
| Fig. 3M | control vs. CCr | *p* = 0.002 |
| Fig. 3M | CCr vs. CCr + Cr | *p* = 0.001 |
| Fig. 3M | control vs. CCr + Cr | *p* = 0.931 |
| Fig. 3N | control vs. Cr | *p* = 0.395 |
| Fig. 3N | control vs. CCr | *p* < 0.001 |
| Fig. 3N | CCr vs. CCr + Cr | *p* < 0.001 |
| Fig. 3N | control vs. CCr + Cr | *p* = 0.202 |
| Fig. 3P | control vs. CCr | *p* = 0.091 |
| Fig. 3P | control vs. CCr + 1 µM GA | *p* = 1 |
| Fig. 3P | control vs. CCr + 5 µM GA | *p* = 1 |
| Fig. 3P | control vs. CCr + 10 µM GA | *p* = 0.271 |
| Fig. 3P | CCr vs. CCr + 1 µM GA | *p* = 0.091 |
| Fig. 3P | CCr vs. CCr + 5 µM GA | *p* = 0.091 |
| Fig. 3P | CCr vs. CCr + 10 µM GA | *p* = 0.498 |
| Fig. 3Q | control vs. CCr | *p* < 0.001 |
| Fig. 3Q | control vs. CCr + 1 µM GA | *p* < 0.001 |
| Fig. 3Q | control vs. CCr + 5 µM GA | *p* = 0.275 |
| Fig. 3Q | control vs. CCr + 10 µM GA | *p* = 0.005 |
| Fig. 3Q | CCr vs. CCr + 1 µM GA | *p* = 0.015 |
| Fig. 3Q | CCr vs. CCr + 5 µM GA | *p* < 0.001 |
| Fig. 3Q | CCr vs. CCr + 10 µM GA | *p* = 0.004 |
| Fig. 3S | control vs. CCr | *p* < 0.001 |
| Fig. 3S | control vs. CCr + 1 µM GA | *p* < 0.001 |
| Fig. 3S | control vs. CCr + 5 µM GA | *p* < 0.001 |
| Fig. 3S | control vs. CCr + 10 µM GA | *p* < 0.001 |
| Fig. 3S | CCr vs. CCr + 1 µM GA | *p* < 0.001 |
| Fig. 3S | CCr vs. CCr + 5 µM GA | *p* = 0.0217 |
| Fig. 3S | CCr vs. CCr + 10 µM GA | *p* = 0.004 |
| Fig. 3T | control vs. CCr | *p* < 0.001 |
| Fig. 3T | control vs. CCr + 1 µM GA | *p* < 0.001 |
| Fig. 3T | control vs. CCr + 5 µM GA | *p* < 0.001 |
| Fig. 3T | control vs. CCr + 10 µM GA | *p* < 0.001 |
| Fig. 3T | CCr vs. CCr + 1 µM GA | *p* = 0.01 |
| Fig. 3T | CCr vs. CCr + 5 µM GA | *p* = 0.258 |
| Fig. 3T | CCr vs. CCr + 10 µM GA | *p* < 0.001 |
| Fig. 3V | control vs. CCr | *p* < 0.001 |
| Fig. 3V | control vs. CCr + 1 µM GA | *p* < 0.001 |
| Fig. 3V | control vs. CCr + 5 µM GA | *p* < 0.001 |
| Fig. 3V | control vs. CCr + 10 µM GA | *p* < 0.001 |
| Fig. 3V | CCr vs. CCr + 1 µM GA | *p* < 0.001 |
| Fig. 3V | CCr vs. CCr + 5 µM GA | *p* < 0.001 |
| Fig. 3V | CCr vs. CCr + 10 µM GA | *p* < 0.001 |
| Fig. 3W | control vs. CCr | *p* < 0.001 |
| Fig. 3W | control vs. CCr + 1 µM GA | *p* < 0.001 |
| Fig. 3W | control vs. CCr + 5 µM GA | *p* < 0.001 |
| Fig. 3W | control vs. CCr + 10 µM GA | *p* < 0.001 |
| Fig. 3W | CCr vs. CCr + 1 µM GA | *p* < 0.001 |
| Fig. 3W | CCr vs. CCr + 5 µM GA | *p* = 0.005 |
| Fig. 3W | CCr vs. CCr + 10 µM GA | *p* = 0.491 |
| Fig. 4C | control vs. Cr | *p* = 0.84 |
| Fig. 4C | control vs. CCr | *p* < 0.001 |
| Fig. 4C | CCr vs. CCr + Cr | *p* < 0.001 |
| Fig. 4C | control vs. CCr + Cr | *p* = 0.584 |
| Fig. 4E | control vs. CCr | *p* = 0.53 |
| Fig. 4E | control vs. CCr + 1 µM Dor | *p* = 0.223 |
| Fig. 4E | control vs. CCr + 5 µM Dor | *p* = 0.548 |
| Fig. 4E | control vs. CCr + 10 µM Dor | *p* = 1 |
| Fig. 4E | CCr vs. CCr + 1 µM Dor | *p* = 0.53 |
| Fig. 4E | CCr vs. CCr + 5 µM Dor | *p* = 0.978 |
| Fig. 4E | CCr vs. CCr + 10 µM Dor | *p* = 0.53 |
| Fig. 4F | control vs. CCr | *p* < 0.001 |
| Fig. 4F | control vs. CCr + 1 µM Dor | *p* = 0.042 |
| Fig. 4F | control vs. CCr + 5 µM Dor | *p* < 0.001 |
| Fig. 4F | control vs. CCr + 10 µM Dor | *p* < 0.001 |
| Fig. 4F | CCr vs. CCr + 1 µM Dor | *p* = 0.001 |
| Fig. 4F | CCr vs. CCr + 5 µM Dor | *p* = 0.066 |
| Fig. 4F | CCr vs. CCr + 10 µM Dor | *p* = 0.54 |
| Fig. 4H | control vs. CCr | *p* < 0.001 |
| Fig. 4H | control vs. CCr + 1 µM Dor | *p* < 0.001 |
| Fig. 4H | control vs. CCr + 5 µM Dor | *p* < 0.001 |
| Fig. 4H | control vs. CCr + 10 µM Dor | *p* < 0.001 |
| Fig. 4H | CCr vs. CCr + 1 µM Dor | *p* < 0.001 |
| Fig. 4H | CCr vs. CCr + 5 µM Dor | *p* = 0.131 |
| Fig. 4H | CCr vs. CCr + 10 µM Dor | *p* = 0.177 |
| Fig. 4I | control vs. CCr | *p* < 0.001 |
| Fig. 4I | control vs. CCr + 1 µM Dor | *p* < 0.001 |
| Fig. 4I | control vs. CCr + 5 µM Dor | *p* < 0.001 |
| Fig. 4I | control vs. CCr + 10 µM Dor | *p* < 0.001 |
| Fig. 4I | CCr vs. CCr + 1 µM Dor | *p* = 0.001 |
| Fig. 4I | CCr vs. CCr + 5 µM Dor | *p* = 0.179 |
| Fig. 4I | CCr vs. CCr + 10 µM Dor | *p* = 0.053 |
| Fig. 4K | control vs. CCr | *p* < 0.001 |
| Fig. 4K | control vs. CCr + 1 µM Dor | *p* < 0.001 |
| Fig. 4K | control vs. CCr + 5 µM Dor | *p* < 0.001 |
| Fig. 4K | control vs. CCr + 10 µM Dor | *p* < 0.001 |
| Fig. 4K | CCr vs. CCr + 1 µM Dor | *p* = 0.002 |
| Fig. 4K | CCr vs. CCr + 5 µM Dor | *p* = 0.384 |
| Fig. 4K | CCr vs. CCr + 10 µM Dor | *p* = 0.301 |
| Fig. 4L | control vs. CCr | *p* < 0.001 |
| Fig. 4L | control vs. CCr + 1 µM Dor | *p* < 0.001 |
| Fig. 4L | control vs. CCr + 5 µM Dor | *p* < 0.001 |
| Fig. 4L | control vs. CCr + 10 µM Dor | *p* < 0.001 |
| Fig. 4L | CCr vs. CCr + 1 µM Dor | *p* < 0.001 |
| Fig. 4L | CCr vs. CCr + 5 µM Dor | *p* < 0.001 |
| Fig. 4L | CCr vs. CCr + 10 µM Dor | *p* = 0.007 |
| Fig. 4O | control vs. Cr | *p* = 0.142 |
| Fig. 4O | control vs. CCr | *p* = 0.002 |
| Fig. 4O | CCr vs. CCr + Cr | *p* = 0.015 |
| Fig. 4O | control vs. CCr + Cr | *p* = 0.243 |
| Fig. 4P (*Hk2*) | control vs. Cr | *p* = 0.714 |
| Fig. 4P (*Hk2*) | control vs. CCr | *p* = 0.048 |
| Fig. 4P (*Hk2*) | CCr vs. CCr + Cr | *p* = 0.026 |
| Fig. 4P (*Hk2*) | control vs. CCr + Cr | *p* = 0.712 |
| Fig. 4P (*Gapdh*) | control vs. Cr | *p* = 0.477 |
| Fig. 4P (*Gapdh*) | control vs. CCr | *p* = 0.185 |
| Fig. 4P (*Gapdh*) | CCr vs. CCr + Cr | *p* = 0.063 |
| Fig. 4P (*Gapdh*) | control vs. CCr + Cr | *p* = 0.50 |
| Fig. 4P (*Pgam2*) | control vs. Cr | *p* = 0.964 |
| Fig. 4P (*Pgam2*) | control vs. CCr | *p* = 0.034 |
| Fig. 4P (*Pgam2*) | CCr vs. CCr + Cr | *p* = 0.026 |
| Fig. 4P (*Pgam2*) | control vs. CCr + Cr | *p* = 0.872 |
| Fig. 4P (*Tpi1*) | control vs. Cr | *p* = 0.986 |
| Fig. 4P (*Tpi1*) | control vs. CCr | *p* = 0.007 |
| Fig. 4P (*Tpi1*) | CCr vs. CCr + Cr | *p* = 0.027 |
| Fig. 4P (*Tpi1*) | control vs. CCr + Cr | *p* = 0.408 |
| Fig. 4P (*Eno1*) | control vs. Cr | *p* = 0.926 |
| Fig. 4P (*Eno1*) | control vs. CCr | *p* = 0.028 |
| Fig. 4P (*Eno1*) | CCr vs. CCr + Cr | *p* = 0.034 |
| Fig. 4P (*Eno1*) | control vs. CCr + Cr | *p* = 0.788 |
| Fig. 4P (*Pkm*) | control vs. Cr | *p* = 0.555 |
| Fig. 4P (*Pkm*) | control vs. CCr | *p* = 0.154 |
| Fig. 4P (*Pkm*) | CCr vs. CCr + Cr | *p* = 0.118 |
| Fig. 4P (*Pkm*) | control vs. CCr + Cr | *p* = 0.866 |
| Fig. 4R (HK2) | control vs. Cr | *p* = 0.338 |
| Fig. 4R (HK2) | control vs. CCr | *p* < 0.001 |
| Fig. 4R (HK2) | CCr vs. CCr + Cr | *p* < 0.001 |
| Fig. 4R (HK2) | control vs. CCr + Cr | *p* = 0.25 |
| Fig. 4R (GAPDH) | control vs. Cr | *p* = 0.725 |
| Fig. 4R (GAPDH) | control vs. CCr | *p* = 0.03 |
| Fig. 4R (GAPDH) | CCr vs. CCr + Cr | *p* = 0.019 |
| Fig. 4R (GAPDH) | control vs. CCr + Cr | *p* = 0.206 |
| Fig. 4R (ENO1) | control vs. Cr | *p* = 0.669 |
| Fig. 4R (ENO1) | control vs. CCr | *p* = 0.01 |
| Fig. 4R (ENO1) | CCr vs. CCr + Cr | *p* = 0.004 |
| Fig. 4R (ENO1) | control vs. CCr + Cr | *p* = 0.532 |
| Fig. 4R (PKM) | control vs. Cr | *p* = 0.832 |
| Fig. 4R (PKM) | control vs. CCr | *p* = 0.775 |
| Fig. 4R (PKM) | CCr vs. CCr + Cr | *p* = 0.782 |
| Fig. 4R (PKM) | control vs. CCr + Cr | *p* = 0.993 |
| Fig. 5B | control vs. Cr | *p* = 0.201 |
| Fig. 5B | control vs. CCr | *p* = 0.045 |
| Fig. 5B | CCr vs. CCr + Cr | *p* < 0.001 |
| Fig. 5B | control vs. CCr + Cr | *p* = 0.093 |
| Fig. 5D | control vs. Cr | *p* = 0.724 |
| Fig. 5D | control vs. CCr | *p* = 0.259 |
| Fig. 5D | CCr vs. CCr + Cr | *p* = 0.819 |
| Fig. 5D | control vs. CCr + Cr | *p* = 0.361 |
| Fig. 5E | control vs. Cr | *p* = 0.62 |
| Fig. 5E | control vs. CCr | *p* = 0.833 |
| Fig. 5E | CCr vs. CCr + Cr | *p* = 0.434 |
| Fig. 5E | control vs. CCr + Cr | *p* = 0.561 |
| Fig. 5G | control vs. Cr | *p* = 0.482 |
| Fig. 5G | control vs. CCr | *p* = 0.035 |
| Fig. 5G | CCr vs. CCr + Cr | *p* = 0.013 |
| Fig. 5G | control vs. CCr + Cr | *p* = 0.675 |
| Fig. 5I | control vs. Cr | *p* = 0.059 |
| Fig. 5I | control vs. CCr | *p* = 0.002 |
| Fig. 5I | CCr vs. CCr + Cr | *p* = 0.41 |
| Fig. 5I | control vs. CCr + Cr | *p* = 0.099 |
| Fig. 5J | control vs. Cr | *p* = 0.424 |
| Fig. 5J | control vs. CCr | *p* < 0.001 |
| Fig. 5J | CCr vs. CCr + Cr | *p* < 0.001 |
| Fig. 5J | control vs. CCr + Cr | *p* = 0.074 |
| Fig. 5K | control vs. 2-DG | *p* = 0.012 |
| Fig. 5M | control vs. 2-DG | *p* < 0.001 |
| Fig. 6H | control vs. Cr | *p* = 0.103 |
| Fig. 6H | control vs. CCr | *p* < 0.001 |
| Fig. 6H | CCr vs. CCr + Cr | *p* < 0.001 |
| Fig. 6H | control vs. CCr + Cr | *p* = 0.022 |
| Fig. 6I | control vs. Cr | *p* = 1 |
| Fig. 6I | control vs. CCr | *p* < 0.001 |
| Fig. 6I | CCr vs. CCr + Cr | *p* < 0.001 |
| Fig. 6I | control vs. CCr + Cr | *p* = 0.002 |
| Fig. 6K | control vs. CCr | *p* = 0.074 |
| Fig. 6K | control vs. 50 µM CK666 | *p* = 0.424 |
| Fig. 6K | control vs. 100 µM CK666 | *p* = 0.414 |
| Fig. 6K | control vs. 200 µM CK666 | *p* = 1 |
| Fig. 6L | control vs. CCr | *p* = 0.001 |
| Fig. 6L | control vs. 50 µM CK666 | *p* = 0.264 |
| Fig. 6L | control vs. 100 µM CK666 | *p* = 0.078 |
| Fig. 6L | control vs. 200 µM CK666 | *p* = 0.002 |
| Fig. 6L | CCr vs. 200 µM CK666 | *p* = 0.03 |
| Fig. 6N | control vs. CCr | *p* < 0.001 |
| Fig. 6N | control vs. 200 µM CK666 | *p* < 0.001 |
| Fig. 6N | CCr vs. 200 µM CK666 | *p* = 0.013 |
| Fig. 6O | control vs. CCr | *p* < 0.001 |
| Fig. 6O | control vs. 200 µM CK666 | *p* < 0.001 |
| Fig. 6O | CCr vs. 200 µM CK666 | *p* = 0.155 |
| Fig. 6P | control vs. CCr | *p* < 0.001 |
| Fig. 6P | control vs. 200 µM CK666 | *p* < 0.001 |
| Fig. 6P | CCr vs. 200 µM CK666 | *p* = 0.032 |

**Table S3. Summary of pairwise comparison *p*-values for supporting figures.**

| **Figure panel** | **Comparison groups** | ***p*-value** |
| --- | --- | --- |
| S1E (morula) | control vs. Cr | *p* = 1 |
| S1E (morula) | control vs. CCr | *p* = 0.44 |
| S1E (morula) | CCr vs. CCr + Cr | *p* = 0.138 |
| S1E (morula) | control vs. CCr + Cr | *p* = 0.428 |
| S1E (96h) | control vs. Cr | *p* = 0.694 |
| S1E (96h) | control vs. CCr | *p* < 0.001 |
| S1E (96h) | CCr vs. CCr + Cr | *p* = 0.002 |
| S1E (96h) | control vs. CCr + Cr | *p* = 0.113 |
| S1E (108h) | control vs. Cr | *p* = 0.489 |
| S1E (108h) | control vs. CCr | *p* < 0.001 |
| S1E (108h) | CCr vs. CCr + Cr | *p* < 0.001 |
| S1E (108h) | control vs. CCr + Cr | *p* = 0.331 |
| S1E (120h) | control vs. Cr | *p* = 0.276 |
| S1E (120h) | control vs. CCr | *p* < 0.001 |
| S1E (120h) | CCr vs. CCr + Cr | *p* < 0.001 |
| S1E (120h) | control vs. CCr + Cr | *p* = 0.436 |
| S1F | control vs. Cr | *p* = 0.03 |
| S1F | control vs. CCr | *p* < 0.001 |
| S1F | CCr vs. CCr + Cr | *p* < 0.001 |
| S1F | control vs. CCr + Cr | *p* = 0.855 |
| S3C | control vs. Cr | *p* = 0.938 |
| S3C | control vs. CCr | *p* = 0.088 |
| S3C | CCr vs. CCr + Cr | *p* = 0.062 |
| S3C | control vs. CCr + Cr | *p* = 0.848 |
| S3K | control vs. Cr | *p* = 0.977 |
| S3K | control vs. CCr | *p* < 0.001 |
| S3K | CCr vs CCr + Cr | *p* < 0.001 |
| S3K | control vs. CCr + Cr | *p* = 0.176 |
| S4B | control vs. Cr | *p* = 0.555 |
| S4B | control vs. CCr | *p* < 0.001 |
| S4B | CCr vs. CCr + Cr | *p* = 0.001 |
| S4B | control vs. CCr + Cr | *p* = 0.002 |
| S4F | control vs. Cr | *p* = 0.548 |
| S4F | control vs. CCr | *p* < 0.001 |
| S4F | CCr vs. CCr + Cr | *p* < 0.001 |
| S4F | control vs. CCr + Cr | *p* = 1 |
| S5B | control vs. Cr | *p* = 0.884 |
| S5B | control vs. CCr | *p* < 0.001 |
| S5B | CCr vs. CCr + Cr | *p* < 0.001 |
| S5B | control vs. CCr + Cr | *p* = 0.395 |
| S5D (72h) | control vs. CCr | *p* < 0.001 |
| S5D (76h) | control vs. CCr | *p* < 0.001 |
| S5D (80h) | control vs. CCr | *p* < 0.001 |
| S6K | Control VS. CK666 | *p* = 0.001 |
| S6M | control vs. Cr | *p* = 0.708 |
| S6M | control vs. CCr | *p* < 0.001 |
| S6M | CCr vs. CCr + Cr | *p* < 0.001 |
| S6M | control vs. CCr + Cr | *p* = 0.708 |
